# Deciphering Microbiome, Transcriptome, and Metabolic Interactions in the Presence of Probiotic *Lactobacillus acidophilus* against *Salmonella* Typhimurium in a Murine Model

**DOI:** 10.3390/antibiotics13040352

**Published:** 2024-04-11

**Authors:** Muhammad Junaid, Hongyu Lu, Ahmad Ud Din, Bin Yu, Yu Liu, Yixiang Li, Kefei Liu, Jianhua Yan, Zhongquan Qi

**Affiliations:** 1 Medical College, Guangxi University, Nanning 530004, China; 2 Plants for Human Health Institute, North Carolina State University, 600 Laureate Way, Kannapolis, NC 28081, USA; 3 Tianjin Shengji Group., Co., Ltd., No. 2, Hai Tai Development 2nd Road, Huayuan Industrial Zone, Tianjin 300384, China

**Keywords:** microbiome, transcriptome, metabolome, immunity, gut barrier integrity, mice, *Salmonella* Typhimurium, *L. acidophilus*

## Abstract

*Salmonella enterica serovar* Typhimurium (*S*. Typhimurium), a foodborne pathogen that poses significant public health risks to humans and animals, presents a formidable challenge due to its antibiotic resistance. This study explores the potential of *Lactobacillus acidophilus* (*L. acidophilus* 1.3251) probiotics as an alternative strategy to combat antibiotic resistance associated with *S*. Typhimurium infection. In this investigation, twenty-four BALB/c mice were assigned to four groups: a non-infected, non-treated group (CNG); an infected, non-treated group (CPG); a group fed with *L. acidophilus* but not infected (LAG); and a group fed with *L. acidophilus* and challenged with *Salmonella* (LAST). The results revealed a reduction in *Salmonella* levels in the feces of mice, along with restored weight and improved overall health in the LAST compared to the CPG. The feeding of *L. acidophilus* was found to downregulate pro-inflammatory cytokine mRNA induced by *Salmonella* while upregulating anti-inflammatory cytokines. Additionally, it influenced the expression of mRNA transcript, encoding tight junction protein, oxidative stress-induced enzymes, and apoptosis-related mRNA expression. Furthermore, the LEfSe analysis demonstrated a significant shift in the abundance of critical commensal genera in the LAST, essential for maintaining gut homeostasis, metabolic reactions, anti-inflammatory responses, and butyrate production. Transcriptomic analysis revealed 2173 upregulated and 506 downregulated differentially expressed genes (DEGs) in the LAST vs. the CPG. Functional analysis of these DEGs highlighted their involvement in immunity, metabolism, and cellular development. Kyoto Encyclopedia of Genes and Genome (KEGG) pathway analysis indicated their role in tumor necrosis factor (TNF), mitogen-activated protein kinase (MAPK), chemokine, Forkhead box O (FOXO), and transforming growth factor (TGF-β) signaling pathway. Moreover, the fecal metabolomic analysis identified 929 differential metabolites, with enrichment observed in valine, leucine, isoleucine, taurine, glycine, and other metabolites. These findings suggest that supplementation with *L. acidophilus* promotes the growth of beneficial commensal genera while mitigating *Salmonella*-induced intestinal disruption by modulating immunity, gut homeostasis, gut barrier integrity, and metabolism.

## 1. Introduction

*Salmonella*, the causative agent of salmonellosis, is a Gram-negative, non-spore-producing foodborne enteropathogenic bacterium [1]. Based on its exceptional combination of surface antigens, it has been classified into 2659 serovars [2]. Among these serovars, *Salmonella enterica* serotype Typhimurium is the second most frequently reported foodborne gastrointestinal infection (GI) in humans after *Campylobacteriosis*, characterized by an immediate onset of fever, abdominal discomfort, vomiting, and diarrhea [3,4,5]. *Salmonella* Typhimurium is generally detected in meats [6,7,8], milk [9], eggs, and their products [10,11,12,13]. Salmonellosis is prevalent in China [14,15,16,17,18]. According to research, *Salmonella* has been found to cause 70–80% of foodborne infections in China [18,19,20]. In February 2022, the UK reported a monophasic *S*. Typhimurium found in chocolate goods produced in Belgium that exhibited rare multi-drug resistance [4]. Intestinal barriers are the first defense against pathogenic organisms and immunogenic substances [21], but *Salmonella* disturbs tight junctions [22]. The gut microbiota, a crucial component of the mucosal intestinal barrier, is essential for maintaining healthy colonic barrier functions [23]. Numerous studies have shown that *Salmonella* infection disturbs gut microbiota homeostasis [24,25], which, in turn, can lead to gut inflammation, providing a nutritional advantage for *Salmonella* growth [24]. Antibiotics have frequently been utilized to treat salmonellosis, while on the other hand, antibiotics consumption produces persistent *Salmonella* [26,27]. Therefore, developing novel and safe strategies for managing and preventing salmonellosis is crucial, with probiotics emerging as a key alternative [28].

Probiotics are living microorganisms that provide health benefits to the host when provided in appropriate amounts, including species like *Lactobacillus*, *Bifidobacterium*, *Saccharomyces boulardii*, *Clostridium butyricum*, and *Bacillus* species [29]. Probiotics have been shown to modulate both adaptive and innate immunities by interacting with immune and epithelial cells, altering the gut microbiota [30], and directly targeting pathogens [31]. They also boost phagocytosis [32,33], and regulate pro- and anti-inflammatory cytokines to protect against gastro-enteric infections [34]. Among these probiotics, *Lactobacillus acidophilus* stands out as a promising contender to influence host–pathogen interactions by coordinating dynamic changes in the host’s microbiome, transcriptome, and metabolome [35,36,37]. It colonizes the gastrointestinal (GI) tract, inhibits the adherence of various enterotoxigenic and entero-invasive bacteria [38], and enhances gut immune barrier functions [39].

This research aimed to comprehensively examine the impact of *Lactobacillus acidophilus* (CGMCC 1.3251) on *S*. Typhimurium infection in a mouse model. We aim to elucidate the intricate mechanisms through which this probiotic can modify the microbiome, transcriptome, and metabolome, potentially altering the outcome of host–pathogen interactions.

## 2. Results

### 2.1. Clinical Observation (Behavioral and Health Parameters)

**Figure 1:**
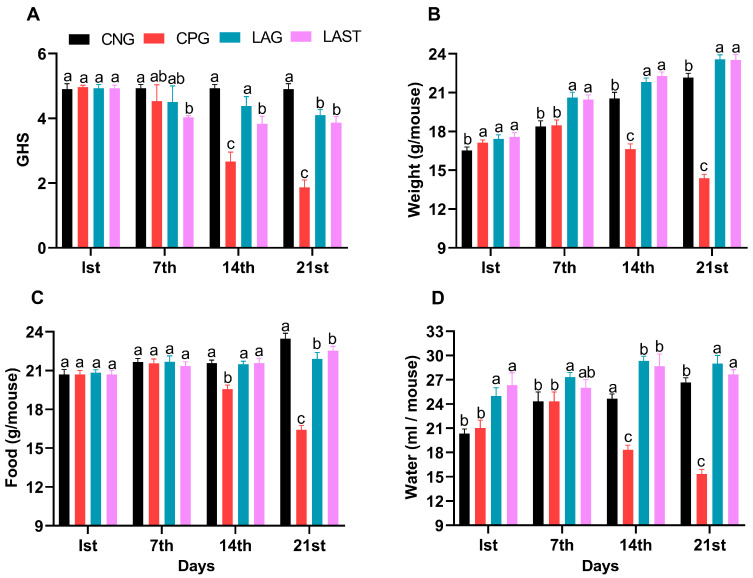
(**A**) General health index score of *Salmonella*-infected and non-infected groups. (**B**) Mean body weight. (**C**) Average food intake. (**D**) Average water intake on day 1, day 7 (infection day), day 14, and day 21. The results were presented with mean ± SD (n = 6). The different letters on the bars represent significant differences between groups in the LSD test, *p* ≤ 0.05.

The general health status of mice in each group was monitored weekly, specifically on day 1 (the first day of the experiment), day 7 (the day of infection), 14 (the first week post-infection), and 21 (the 2-week post-infection). The *Salmonella*-infected non-treated group (CPG, positive control) demonstrated visual clinical symptoms of illness and lower health index post-infection compared to the non-infected non-treated group (CNG), the *Lactobacillus acidophilus*-fed non-infected group (LAG), and the *Lactobacillus acidophilus*-fed *Salmonella*-challenged group (LAST) (Figure 1A). The mean body weight of each mouse was also recorded on alternative days (1, 7, 14, and 21). Before the *Salmonella* infection, each group’s body weight curve trend was similar from day 1 to 7. Over the 21 days of the experiment, average weight gain was recorded in CNG and LAG. Conversely, the CPG exhibited weight loss post-infection, while the *Salmonella*-challenged but treated with *L. acidophilus* group (LAST) recovered and showed weight gain compared to the CPG (Figure 1B). Additionally, food and water intakes were also assessed weekly. As compared to CNG, the *Salmonella*-challenged but non-treated group, i.e., CPG, showed a moderate decrease in food and water intakes after infection, while the probiotic group (LAG) and the challenged but treatment group (LAST) maintained the food and water intakes (Figure 1C,D).

#### Ambulation, Grasping Reflex, and Fecal Salmonella Count

**Figure 2:**
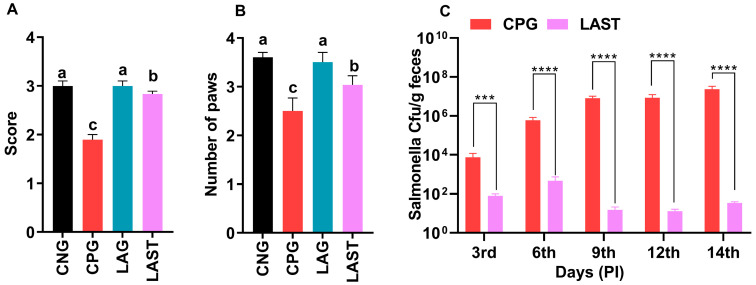
Strength-associated defects interceded by *Salmonella*. (**A**) Ambulation (10 dpi). (**B**) Grasping reflex (10 dpi). Data are presented as ± SD from three replicates. Different letters on the bars show significant differences in the LSD test (*p* < 0.05). (**C**) Viable *Salmonella* counts in CPG and LAST feces on alternative days post-infection. Data are presented as log_10_ CFU/g of feces. Asterisk indicates significant differences between CPG and LAST using a *t*-test (*** *p* < 0.001, **** *p* < 0.0001).

The results revealed that the *Salmonella*-infected but non-treated group, i.e., CPG, exhibited lower ambulation scores than CNG and LAG. However, these adverse effects were significantly mitigated following treatment with *L. acidophilus* in the LAST (Figure 2A). Additionally, the grasping reflex test showed that mice in the CPG displayed defective posterior paw reflexes, whereas *L. acidophilus* treatment (LAST) significantly improved the mice from the rear foot grasping flaws (Figure 2B).

Unlike CPG and LAST, no viable *Salmonella* was detected in the negative control group (CNG) and probiotic group (LAG). Furthermore, compared to CPG, mice challenged with *Salmonella* but fed *Lactobacillus acidophilus* for seven days before infection exhibited reduced *Salmonella* counts in feces when measured on days 9, 12, and 14 post-infection. These findings demonstrated that animals administered the probiotic *L. acidophilus* (LAST) had a lower colony-forming unit (CFU) of *Salmonella* compared to the positive control group (CPG) (Figure 2C). 

### 2.2. mRNA Expression Levels of Different Cytokines

#### 2.2.1. Pro- and Anti-Inflammatory Cytokine Expression

**Figure 3:**
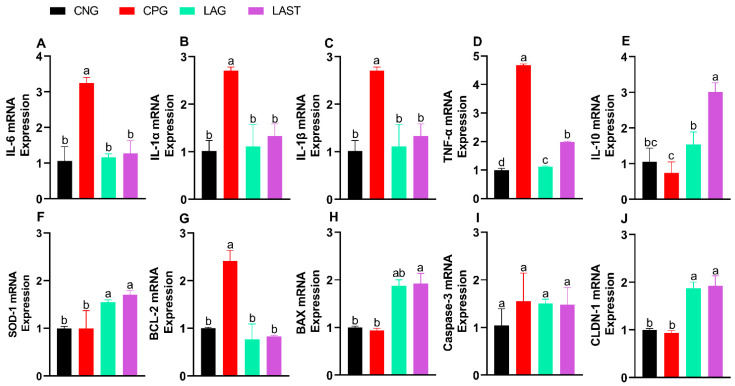
Effect of *L. acidophilus* on relative expression of mRNA of different genes: (**A**) IL-6, (**B**) IL-1α, (**C**) IL-1β, (**D**) TNF-α, (**E**) IL-10, (**F**) SOD-1, (**G**) BCL-2, (**H**) BAX, (**I**) CASPASE-3, and (**J**) CLDN-1. The results are presented as mean ± SD (n = 3). The bar above the column indicates a standard error, and the different letters show significant differences among groups in the LSD test (*p* ≤ 0.05).

The *Salmonella* infection significantly altered mRNA levels of pro-inflammatory cytokines (IL-6, IL-1α, IL-1β, and TNF-α). Specifically, the CPG showed upregulation, while LAST and LAG downregulated their expression (Figure 3A–D) (*p* ≤ 0.05). Additionally, IL-10 expression significantly improved in the LAST as compared to that in CPG (Figure 3E) (*p* ≤ 0.05).

#### 2.2.2. Antioxidant mRNA Expression

The SOD-1 mRNA expression level was also evaluated. The group, CPG, demonstrated a reduction in SOD-1 expression, while LAST and LAG slightly upregulated their expression (Figure 3F).

#### 2.2.3. Apoptosis-Related Gene Expression

Figure 3G,H depict the mRNA expression pattern of apoptosis-related genes. The CPG downregulated the Bax while upregulated the Bcl-2 expression compared to those of LAG and LAST. However, the expression of caspase-3 mRNA remained relatively consistent across CNG, LAG, and LAST as compared to that of the CPG treatment (Figure 3I).

#### 2.2.4. Tight Junction Proteins

In the group challenged with *Salmonella* but not treated (CPG), the mRNA expression level of CLDN1 exhibited downregulation, whereas LAG and LAST treatments significantly upregulated their expression (Figure 3J).

### 2.3. Alpha and Beta Diversities

The alpha diversity metrics including Ace, Chao1, Shannon, and Simpson were observed. Their significance was validated by the Kruskal–Wilcox test, but no significant differences were found (Figure S1A–D). The rarefaction curve demonstrated the sufficient richness of the detected OTU and the sequencing depth (Figure S2). Beta diversity was analyzed using non-metric multidimensional scaling (NMDS) and principal coordinates analysis (PCoA) with Bray–Curtis distances to assess fecal microbiota differences between groups. The PCoA and Anosim (analysis of similarity) plots based on Bray–Curtis distance indicated significant differences in the gut microbiota between groups, reflecting the influence of *L. acidophilus* or *Salmonella* on the gut microbial structure. The results showed that bacterial communities in each group exhibited distinct patterns (*p* = 0.04). Furthermore, PCoA1 and PCoA2 accounted for 31.7% and 19.1% variance in bacterial communities (Anosim, R = 0.42) (Figure S3A).

NMDS further confirmed dissimilarities among groups and significant matrices (Figure S3B). Furthermore, hierarchical clustering analysis divided samples into two main groups, one containing LAG samples while the rest of the samples further subdivided into several groups (Figure S4).

### 2.4. OTU Analysis

**Figure 4:**
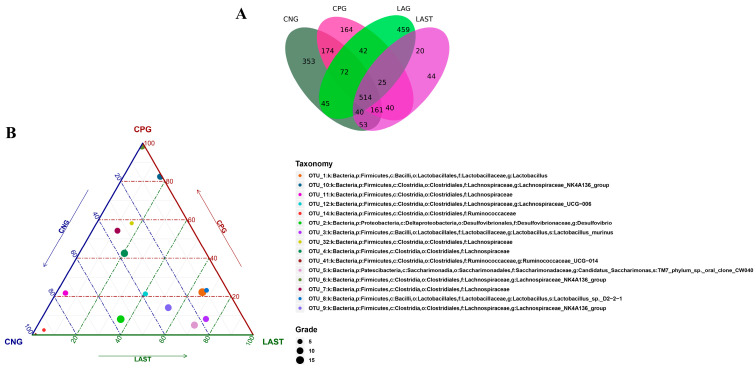
(**A**) Venn diagram analysis represents each group’s total and unique OTU numbers. (**B**) Ternary plot analysis displaying the enriched and depleted genera for bacterial community composition between groups: CNG, CPG, and LAST.

All sequences were categorized into OTUs based on the different levels of similarities, with each OTU corresponding to a typical sequence. The treatment group has 514 common OTUs, while 353,164,459 and 44 unique OTUs were detected in CNG, CPG, LAG, and LAST, respectively (Figure 4A). Meanwhile, the ternary phase diagram showed a higher abundance of *Lactobacillus*, *Bacilli*, *Lactobacillus murinus*, *Proteobacteria*, and *Desulfovibrio* in the LAST. In contrast, CPG showed a higher abundance of *Clostridial* and some *Lachnospiriaceae* groups. Furthermore, the CNG exhibited an abundance of *Lachnospiriaceae* and *Ruminicoccaceae* families (Figure 4B).

#### 2.4.1. Effects of Treatment on Bacterial Abundance on Phylum and Genus Levels

**Figure 5:**
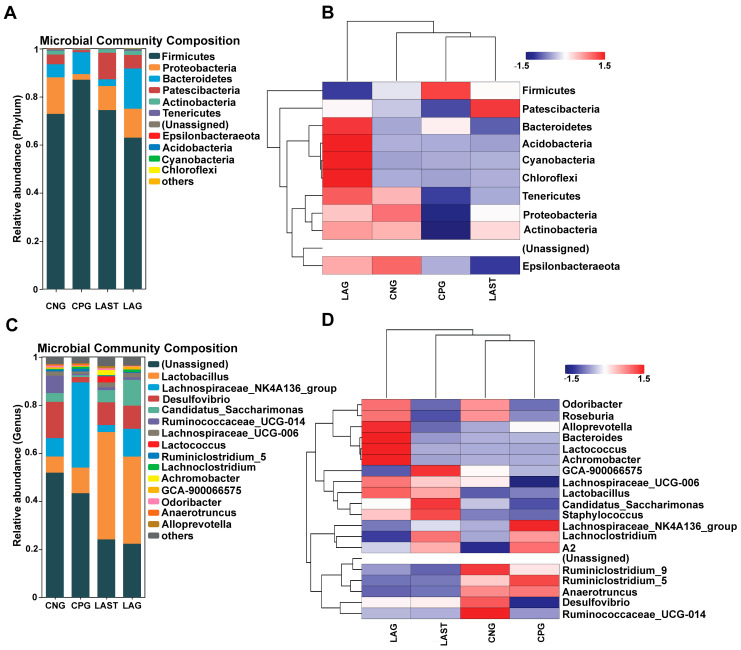
(**A**) Relative abundance of taxa at the phylum level. (**B**) Hierarchical heatmap displaying the richness variation in gut microbes at the phylum level amongst the groups. (**C**) Taxa richness at the genus level among all groups. (**D**) Hierarchical heatmap clustering displaying the abundance alteration in gut–microbiota at the genus level (Top 15). Red color indicates high abundance, white color indicates average abundance, and blue color indicates low abundance.

At the phylum level, the abundance of *Firmicutes* was predominant in all four groups: CNG (72%), CPG (87%), LAG (62%), and LAST (74%), respectively. However, *Proteobacteria* and *Patescibacteria* were significantly affected in the CPG (2%, 0.8%) compared to the CNG (15%, 4%), while the probiotic *L. acidophilus*-treated, *Salmonella*-infected group, LAST, restored its abundance (10%, 11%). Additionally, LAG showed a higher abundance of *Bacteriodetes*, *Acidobacteria*, *Cyanobacteria*, and *Chloroflexi* (Figure 5A,B). At the genus level, CPG had a significantly higher *Lachnospiraceae_NK4A136* (35%) than CNG (7.6%), LAG (2%), and LAST (11%). Additionally, CPG had a decreased abundance of *Desulfovibrio* (15%) and *Ruminococcaceae_UCG-014* (7.1%), which were restored by LAG (9.4%, 1.1%) and LAST (9.6%, 1%). Notably, CPG had a decreased abundance of *Lactobacillus* (10%), while LAST and LAG increased their abundance to 44% and 36%, respectively (Figure 5C,D).

#### 2.4.2. LEfSE Analysis

**Figure 6:**
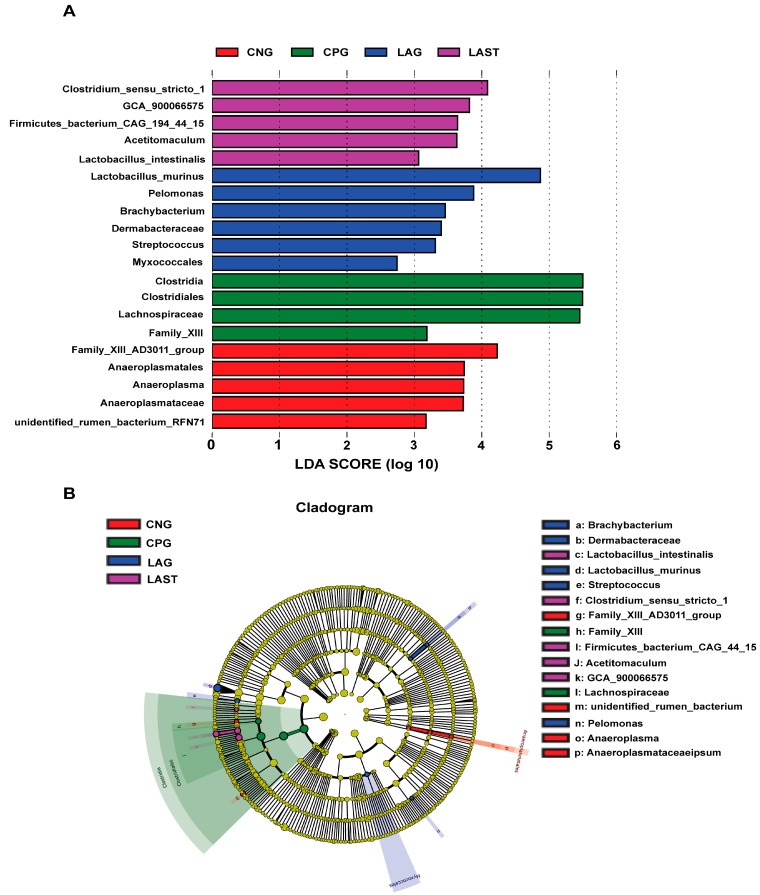
Intestinal microbiota biomarkers among groups. (**A**) LEfSe analysis showed differentially abundant taxa produced by the Kruskal–Wallis and the Wilcox test. (**B**) Cladogram of taxa abundances between groups. The taxa lacking significant differences are labeled in yellow, whereas significantly diverse taxa employ the color of the individual group; red indicates the control negative group (CNG), green indicates the control positive group (CPG), blue represents the probiotic group (LAG), and purple represents the treatment group (LAST). Taxa with a log-linear discriminant analysis (LDA) score of >2 were finally considered (*p* ≤ 0.05).

The LEfSe analysis showed a significant microbial shift due to *Salmonella* infection and probiotic treatment, as demonstrated by bar charts and a cladogram (LDA ≥ 2). The effect size (LEfSe) algorithm identified the genera *Clostridium*, *Tyzzerela*, *Achromobacter*, *Leucobacter*, *Burkholderia*, *Lachnospira*, *Sphingomonas*, and *Coprococcus_3* as abundant in CPG (*p* ≤ 0.05). Conversely, LAST significantly elevated the abundance of *Enterococcus*, *Staphylococcus*, *Clostridium*, *Enterorhabdus*, *Candidatus-saccharimonas*, and *Acetitomaculum* (*p* ≤ 0.05) (Figure S5A,B). Further comparing all four groups, LAST showed enriched *Lactobacillus* and *Acetitomaculum*, CPG had *Clostridia*, LAG had *Pelomonas* and *Brachybacterium*, and CNG had *Anaeroplasma* (*p* ≤ 0.05) (Figure 6A,B). The groups CNG vs. CPG and CNG vs. LAG were also analyzed and are presented in Supplementary Materials (Figures S6A,B and S7A,B).

#### 2.4.3. Spearman Correlation and Redundancy Analysis

**Figure 7:**
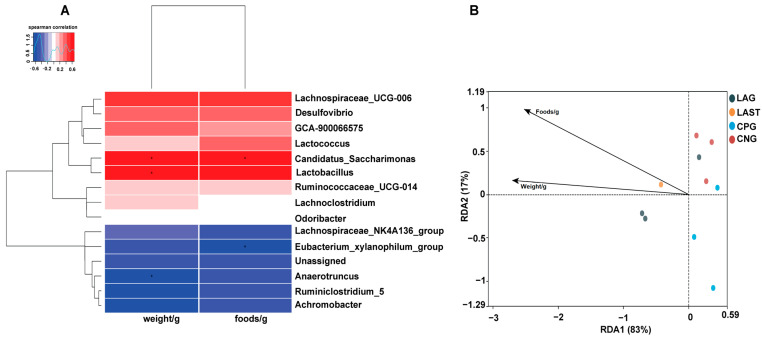
(**A**) Hierarchical heatmap of Spearman correlation between environmental factors and top 15 bacterial genera. (**B**) Distance-based RDA analysis showing the association between gut microbiota variations and ecological variables. Data are presented as the mean ± SD (n = 3). * shows the significance (*p* < 0.05).

Figure 7A illustrates the Spearman correlation among various environmental factors like food and weight with the top 15 abundant bacterial genera. Most parameters were positively associated with the main bacterial species such as *Lactobacillus* and *Candidatus_saccharimonas* while *Eubacterium_xylanophilum* group and *Anaerotruncus* showed negative correlations. Furthermore, the redundancy analysis (RDA) showed that the first two quadrants enlightened 100% disparity between the samples, with RDA1 explaining 83% and RDA2 explaining 17% of the total variation (Figure 7B).

### 2.5. Transcriptomics Data Analysis

To investigate the potential molecular mechanisms of the probiotic *L. acidophilus* in preventing *Salmonella* infection, we performed 3′ mRNA sequencing on small intestinal (ileum) tissue samples from three groups: CNG (control negative group), CPG (*Salmonella*-infected, untreated), and LAST (*Salmonella*-infected, *L. acidophilus*-treated). Nine intestinal libraries were generated and sequenced, yielding 94 million raw reads. After filtering and trimming, 85.2 million clean reads and 16.65 G clean bases were obtained. The average Q20 score was 98.3%, the Q30 score was 94%, and the average GC content was 44.1% (Table S4).

#### 2.5.1. Differentially Expressed Genes Influenced by *Salmonella* Typhimurium and *Lactobacillus acidophilus* Exposures

**Figure 8:**
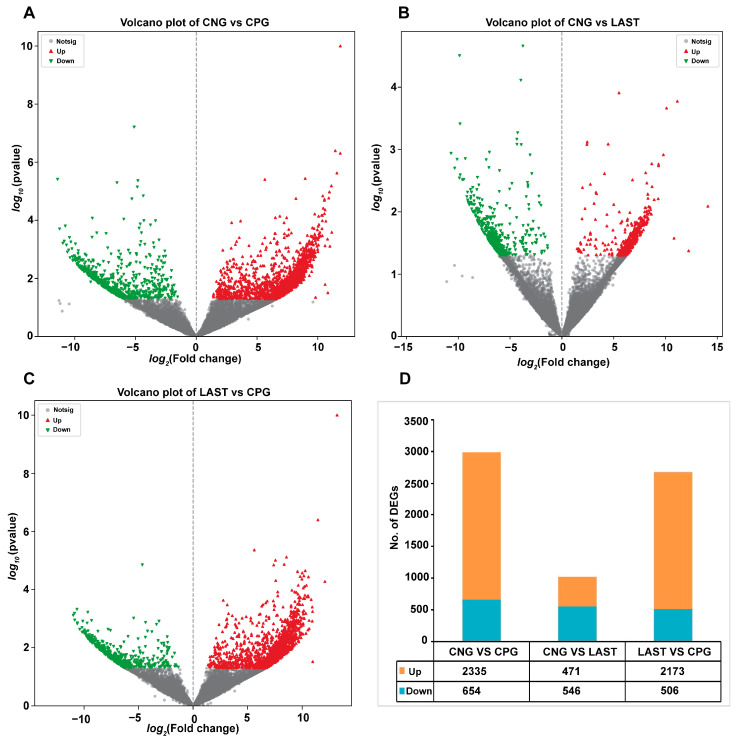
Volcano plot of differentially expressed upregulated and downregulated genes between groups: (**A**) CNG vs. CPG, (**B**) CNG vs. LAST, (**C**) LAST vs. CPG, and (**D**) summary of DEGs. Red color indicates upregulated DEGs, green color indicates downregulated DEGs, and gray color refers to non-significant DEGs.

In total, 2335 upregulated and 654 downregulated DEGs were detected in the CNG vs. CPG, 471 upregulated and 546 downregulated DEGs were found in CNG vs. LAST, and the LAST vs. the CPG showed 2173 upregulated and 506 downregulated DEGs (Figure 8A–D). When exposed to *L. acidophilus*, LAST demonstrated an increase in the expression of various immunity-related genes, including CXCL10, CHCL11, TLR13, CCL22, CCL28, interleukins (IL18, IL1ral1, IL22ra2), Tab1, Tab3, and Cd9 antigen. Genes associated with cell apoptosis and regulation (Casp8 and Bcl2l15) were also elevated. Furthermore, genes involved in gut barrier integrity (CLDN-1, CLDN-2, TJP1, and OCLN) showed increased expression. Finally, some metabolism-related genes (AGPAT4, UCK2, and AGPAT5) were also elevated in LAST compared to CPG (Table S5).

#### 2.5.2. Gene Ontology Annotation Analysis for Unique Differentially Expressed Genes

The gene ontology (GO) enrichment analysis was conducted on differentially expressed unique genes between the LAST (*Salmonella*-challenged, *L. acidophilus*-treated) and CPG (*Salmonella*-challenged, untreated) groups to elucidate the genetic mechanisms underlying probiotic *L. acidophilus* supplementation and *Salmonella* infection. The DEGs were classified into functional groupings using the GO classification system, including biological processes (BP), cellular components (CC), and molecular functions (MF). In the LAST vs. CPG comparison, 1377, 1519, and 1409 GO terms were associated with upregulated DEGs, while 310, 330, and 306 GO terms were linked with downregulated DEGs (Table S6).

**Figure 9:**
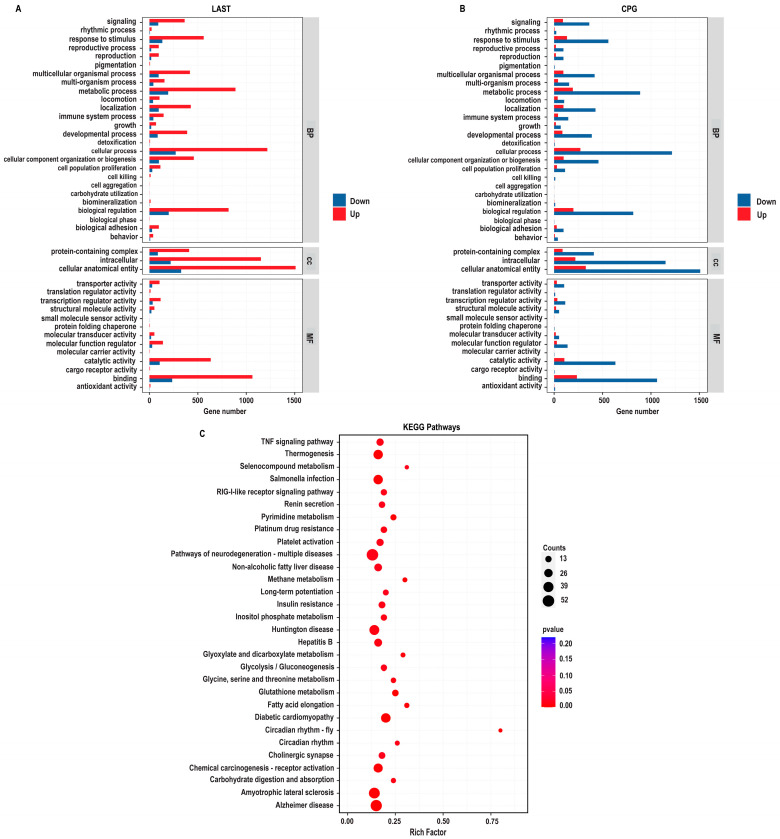
GO enriched terms and KEGG enrichment pathway analysis of upregulated and downregulated DEGs in LAST vs. CPG. (**A**) Upregulated GO enriched terms, classified into BP, biological process, CC, cellular component, and MF, molecular function, in LAST. (**B**) Downregulated GO-enriched terms, classified into BP, biological process, CC, cellular component, and MF, molecular function, in CPG. (**C**) Top 30 KEGG-enriched pathway analyses (*p* ≤ 0.05).

The enriched GO-BP that is elevated in the LAST compared to the CPG encompassed processes related to the immune system, localization, pigmentation, reproduction, signaling, biogenesis, and metabolic processes. The GO-CC described complex, intracellular, and cellular anatomical entities containing proteins. At the same time, the enriched GO-MF terms included binding activity, catalytic activity, structural molecular activity, and transcriptional and translational regulation activities (Figure 9A).

#### 2.5.3. Comprehensive KEGG Pathway Analysis of DEGs

To determine the impact of probiotic *L. acidophilus* supplementation, enrichment pathway analysis was plotted by mapping DEGs to the KEGG database. The most significant enriched pathways in the LAST compared to the CPG included the TNF signaling pathway, RIG-I-like receptor signaling pathway, thermogenesis, circadian rhythm, pyrimidine metabolism, methane metabolism, platelet activation, glycine, serine, and threonine metabolism, glutathione metabolism, seleno-compound metabolism, carbohydrate digestion and absorption, glycolysis, inositol phosphate metabolism, MAPK signaling pathway, TGF-beta signaling pathway, FOxO signaling pathway, Rap1 signaling pathway, and chemokine (Figure 9B and Table S7).

### 2.6. Effects of Lactobacillus acidophilus and Salmonella Typhimurium on Metabolomics in Intestinal Contents, PCA, and OPLS-DA Analysis

Non-targeted LC-MS analysis was employed to compare the LAST and CPG metabolite profiles. The PCA analysis revealed distinct sample separation, with PC1 accounting for 46.77% and PC2 accounting for 21.13% of the total variance. Furthermore, the OPLS-DA model for LAST vs. CPG revealed substantial differences, with R2X(cum) = 0.596, R2Y(cum) = 0.996, and Q2(cum) = 0.687 (Figure S8A–D).

#### 2.6.1. Differential Metabolite Screening between Groups

**Figure 10:**
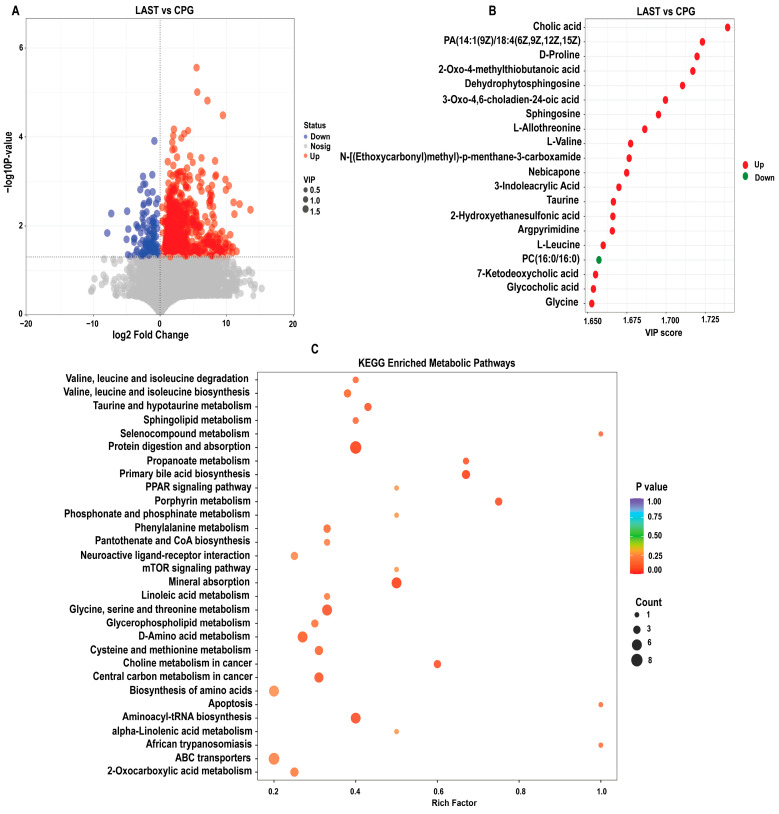
(**A**) Volcano plot showing significantly expressed upregulated and downregulated metabolites between LAST vs. CPG. (**B**) Top 20 significant metabolites showing a variable importance in projection (VIP ≥ 1) score in LAST vs. CPG. (**C**) Top 30 KEGG pathway significantly enriched by differentially abundant metabolites between LAST vs. CPG, (*p* ≤ 0.05). Red color indicates the upregulated metabolic pathways, and blue color indicates the downregulated metabolic pathways, and bubble dot size shows the abundance of enrichment.

To evaluate the influence of probiotic *L. acidophilus* on *Salmonella* and intestinal metabolites in LAST vs. CPG. A total of 929 significantly altered metabolites, with 747 upregulated and 182 downregulated were identified (Figure 10A and Table S8). The top 20 metabolites meeting the criteria of VIP ≥ 1, *p* ≤ 0.05, and log 2FC > 1 included cholic acid, D-proline, dehydro-phytosphingosine, L-allothreonine, L-valine, nebicapone, 3-indole acrylic acid, taurine, argpyrimidine, 7-ketodeoxycholic acid, glycocholic acid, L-leucine, and argpyrimidine (Figure 10B). Furthermore, hierarchical cluster analysis (HCA) highlighted significantly upregulated and downregulated metabolites in LAST vs. CPG, such as L-alanine, nebicapone, L-urobilin, 4-acetylbutyrate, L-threonine, afalanine, N-acetyl-L-alanine, choline, 2-hydroxybutyric acid, sphingosine, 2-(methylthio) propane, glycine, glycocholic acid, DL−3-phenyllactic acid, phenethanolamine, taurine, L-tryptophan, D-phenylalanine, and others (Figure S10).

#### 2.6.2. KEGG Metabolic Pathway Analysis of Differential Metabolites

The KEGG pathway analysis was conducted on the highly upregulated metabolites in the LAST vs. CPG comparison. This approach contributes to understanding the mechanisms behind metabolic alterations in various experimental samples. Several essential pathways were significantly enriched (*p* < 0.05), including valine, leucine, and isoleucine degradation, taurine and hypo-taurine metabolism, aminoacyl-tRNA biosynthesis, ABC transporters, amino acid biosynthesis, glycine, serine, and threonine metabolism, apoptosis, mTOR signaling pathway, PPAR signaling pathway, protein digestion and absorption, porphyrin metabolism, mineral absorption, and phenylalanine metabolism (Figure 10C).

## 3. Discussion

Our bodies are frequently exposed to infection by many dietary pathogens in our daily lives, such as *Salmonella*, which can induce host enterocolitis, characterized by severe intestinal inflammation, impaired absorption of nutrients, a compromised immune system, and bloody diarrhea [40,41]. *Salmonella* is a leading public health concern worldwide due to its emerging resistance to antibiotics and its burden on the health system leads to 93.8 million cases and a death toll of 155,000 annually [42]. *Salmonella* treatment has historically relied on conventional antibiotic therapies, yet rising antibiotic resistance demands the adoption of alternative approaches, with probiotics emerging as a key consideration. Considering the role of probiotics as an emerging treatment for various diseases, several studies have been conducted to evidence their role in the treatment of various diseases specifically against enteropathogenic microorganisms [43]. In the treatment of enteropathogenic diseases, probiotics help maintain gut homeostasis [44,45] and gut barrier integrity [46], enhance nutrient digestion [47], and defend against pathogens by modulating immune responses [48]. Building upon these probiotic mechanisms, the analyses of the microbiome, transcriptome, and metabolome were conducted to investigate the beneficial effects of probiotic *L. acidophilus* in response to the detrimental effects of *Salmonella* infection.

The results of this study revealed that the GHS score of the treated group (LAST) was maintained compared to the *Salmonella*-infected group (CPG), confirming that *Salmonella* infection caused a reduced food intake, gastrointestinal problems, and an altered physiological condition that contribute to weight loss, a clinical sign of *Salmonella* infection, while *L. acidophilus* has a positive impact on the GHS. Probiotics play a key role in the regulation and modulation of immune response, and in turn, they provide promising evidence for a better food intake and improved health cognition and immunity by regulating the gut microbiota [30,49]. Adding *L acidophilus* significantly influenced the GHS markers by enhancing the food intake and nutrient absorption and cornered the effect triggered by the *Salmonella* infection. It was suggested that supplementing with the probiotic *L. acidophilus* could prevent weight loss induced by the *Salmonella* challenge [50]. Consistent with these findings, the current result revealed that the GHS was low in CPG, while the group infected with *Salmonella* but treated with *L. acidophilus* (LAST) reversed the adverse effect and showed a higher GHS. The probiotic *L. acidophilus*-fed mice maintained considerably greater average food and water consumption and experienced increased weight throughout the first 14 days following infection, as evidenced in their behavioral patterns (Figure 1A–D). These findings suggest that *L. acidophilus* significantly attenuated the severity of infection in mice exposed to *Salmonella* or might be blocking its attachments to the cell and interfered with their invasion into the cell by competing with the S-layer protein, lipoteichoic acid, and extracellular polysaccharide of the pathogenic bacteria [51,52]. The above results are in parallel with those of the study by Maia et al. [53], indicating that *L. acidophilus* significantly increased the survival time of mice challenged with *S*. Typhimurium. On the other hand, the adverse effect of *Salmonella* infection resulted in numerous organ failures and fatigue, accounting for strength-related problems [54]. The behavioral assessments, including grip strength and ambulation tests, clearly demonstrated that *Salmonella*-infected mice in the CPG were weaker in strength, as observed in the current study. Conversely, treatment with *L. acidophilus* helped restore mice from infection and associated strength-related impairments (Figure 2A,B). A similar outcome was reported by Naik et al. [54], revealing that mice treated with *Lactobacillus rhamnosus GG* exhibited improved ambulation and grasping reflexes.

Probiotics play a significant role in protecting against intestinal pathogens like *S*. typhimurium through various mechanisms, one of which is immune modulation. In salmonellosis, there is often an increase in levels of pro-inflammatory cytokines like interleukins (IL-1 and IL-6), TNF-α, and interferon-gamma (IFN-γ). These cytokines create an inflamed environment that is conducive to the multiplication and pathogenicity of *Salmonella* while also disrupts the components of the indigenous microbiota [55]. The results of this study provide sufficient evidence of the increase in inflammatory cytokines due to the *Salmonella* infection. In the CPG, higher levels of mRNA transcript for pro-inflammatory cytokines (IL-1α, IL-1β, IL-6, and TNF-α) were found, while the treatment group, LAST, significantly normalized their expression. Furthermore, the IL-10 mRNA expression level in the LAST was higher than that in the CPG (Figure 3A–E). Following infection, the consistent administration of probiotics both before and after exposure to *Salmonella* helped protect the host by regulating the inflammatory response, particularly within the immune effector site of the gut. This regulation involved a reduction in TNFα levels and an increase in the production of IFNγ and IL-10 within the small intestine [56,57], which is consistent with the study of Zhendong Cai et al. [58], as *L. acidophilus CICC 6074* significantly reduced the inflammatory cytokines such as TNF-α, IL-1α, IL-1β, IFN-α, and IFN-β in mice. Antioxidant activity was also assessed, revealing that the probiotic-treated group (LAST) exhibited a higher mRNA expression of SOD-1 than the untreated CPG (Figure 3F). *Salmonella* generates reactive oxygen species (ROS) as a part of its pathogenic mechanism, causing oxidative damage to the host cells [59], while superoxide dismutase-1 (SOD-1) functions as an antioxidant enzyme, facilitating the conversion of superoxide into the relatively less harmful substances, i.e., hydrogen peroxide and oxygen, through a process known as dismutation [60]. Parallel studies were found by El Jakee et al. [61], as *L. gasseri* and *L. casei* exhibited a significant antioxidant activity in mice compared to the untreated group. Apoptosis caused by *S*. Typhimurium infection may lead to intestinal damage [62]. Probiotics have increased caspase and Bax mRNA expression while decreasing anti-apoptotic Bcl-2 levels [63]. These findings indicate that mice administered with probiotics (LAG and LAST) boosted the Bax mRNA expression while reducing BCL-2 expression compared to CPG. However, no significant changes in caspase-3 expression were found (Figure 3G–I), aligning with the study conducted by F.U Memon et al. [64]. Similarly, another study found an increase in the expression of Bax, p53, and caspase 3 genes, along with a decrease in Bcl2, TNF-α, and IL-6 genes, observed in both the intestines and the lungs of rats treated with a mixture of probiotics compared to those treated with heavy metals [65]. Furthermore, *S*. Typhimurium infection can disrupt the intestinal tight junction (ITJ) by decreasing mucus layer and TJ protein expression [66]. The intestine is crucial for combining immune response with the processes of nutrient digestion and absorption. Tight junctions (TJs) formed by neighboring intestinal epithelial cells are vital components of the intestinal barrier, controlling the passage of ions, solutes, water, and other substances through the intestinal epithelium [67]. Claudins (CLDNs) primarily facilitate the connection between neighboring enterocytes by interacting with their extracellular loops within tight junction (TJ) complexes [68]. The results revealed that probiotic feeding enhanced the CLDN1 expression in the treated groups LAG and LAST, while the expression level was mitigated in the *Salmonella*-infected CPG mice (Figure 3J). The above results are in line with the findings of Lihong Wang et al. [69], who showed that *L. plantarum LTC-113* protects host from *Salmonella*-induced intestinal barrier disruption in newly hatched chickens. This study is also in parallel with the findings of Zhang et al. [70], who showed that *B. subtilis* LF11 significantly upregulated the transcription levels of tight junction CLDN1 genes in *Salmonella*-infected NCM460 cells.

One of the prominent features of intestinal diseases is gut microbiota dysbiosis [25]. It was observed that *L. acidophilus* did not affect the alpha diversity significantly. However, beta diversity analysis showed alterations among groups (Figure S3A,B). A total change of 50.8% was observed, with PCoA1 accounting for 31.7% and PCoA2 for 19.1% of the total variation. These observed changes confirm a significant difference at the beta diversity level. Gaining insights into how *Salmonella* infection alters the host microbiome is crucial for enhancing our comprehension of *Salmonella* mechanisms of pathogenesis and disease progression [71]. Due to *Salmonella* infection, some studies suggest that the infection may lead to the alterations in *Firmicutes*, *Proteobacteria*, and *Bacteroidetes* at the phylum level and *Lactobacillus* at the genus level [72]. In the current study, it was observed that the *Salmonella*-infected mice CPG had reduced *Proteobacteria* and *Patescibacteria*, while CNG, LAG, and LAST maintained their abundance (Figure 5A–D). A study by Bescucci et al. [73] reported that infection with *Salmonella* leads to a decrease in the abundance of *Proteobacteria* and *Patescibacteria*, confirming the reliability of our samples and analytical methods. Furthermore, the LEfSe analysis revealed that LAST exhibited a significant abundance in important genera like *Enterococcus*, *Staphylococcus*, *Clostridium_sensu_stricto_1*, *Bacillales*, *Enterorhabdus*, *Candidatus-saccharimonas*, *Staphylococcus*, *Acetitomaculum*, and *Lactobacillus intestinalis* compared to CPG (LDA > 3). Conversely, CPG showed abundance in *Clostridiales*. The LAG showed abundance in *Lactobacillus_murinus*, *Pelomonas*, *Dermabacteraceae*, and *Streptococcus* (LDA > 3), whereas *Anaeroplasma* showed abundance (LDA > 3) in the CNG (Figure 6A,B and Figures S5–S7). The host gut microbiota is crucial in regulating immunity and metabolic pathways, reducing inflammation during enteric infection [74]. Increased *Lactobacillus* can improve intestinal barrier defense and restore the gut microbiota [75,76,77,78]. Genus *Clostridium sensu_stricto_1* facilitated butyrate production, which helps mitigate inflammation, shield the epithelial barrier, and modify colonic motility [79]. Furthermore, Biswaranjan et al. [80] showed the effectiveness of *L. acidophilus* in ameliorating the microbial dysbiosis and inflammation caused by *Salmonella* infection in Th1- and Th2-biased mice. *Enterococcus* species serve as probiotics, producing anti-microbial peptides [81]. *Bacillus* and *Staphylococcus* aid in metabolism and host health maintenance [82]. *Candidatus Saccharimonas* members influence inflammatory diseases and modulate immune responses [83]. Research has linked specific *Lactobacillus* and *Candidatus* species to weight gain [84], which aligns with our findings.

Utilizing gene expression array technology significantly enhances our comprehension of host–pathogen interactions, particularly in the context of *Salmonella* infection. While previous genomics research has identified numerous genes responsive to *Salmonella* infection, there is still much to be explored in this field [85]. In this study, the DEGs in the LAST indicated upregulated genes linked to immunity, homeostasis, gut integrity, apoptosis, and metabolism compared to the CPG (Table S5), consistent with the prior research demonstrating probiotics’ influence on DEGs linked to immunity, homeostasis, gut integrity, apoptosis, and metabolism [37,86,87]. By utilizing GO and KEGG analyses, we investigated the roles and interconnections of differentially expressed genes (DEGs) within biological pathways. When comparing treated LAST to the untreated CPG, we identified enriched GO keywords and KEGG pathways associated with immunity, barrier integrity, and metabolism. These pathways include TNF signaling, RIG-I-like receptor signaling, MAPK signaling, TGF-β signaling, chemokine signaling, platelet activation, and FoxO signaling as well as pathways related to glycine, serine, and glutathione metabolism. Additionally, pathways such as inositol phosphate metabolism and Rap1 signaling pathway were among the most significantly enriched KEGG pathways (Figure 9B). Key pathways such as MAPK, TNF, chemokine, and RIG-I-like receptor signaling, cellular communication, and tissue homeostasis, including immune cell activation (T-cells, B-cells, and macrophages), cytokine production, and phagocytosis, were significantly influenced by probiotic treatment [84]. The TGF-β and FoxO signaling pathways play multiple roles in cell development, differentiation, apoptosis, and immunity [88]. Similarly, Huang et al. [89] demonstrated that cells pretreated with *L. acidophilus* or symbiotic compared to *S*. Typhimurium-infected cells significantly elevated the expression of the TGF-β1 signaling pathway, which may be involved in the inflammation-suppressive effects of *L. acidophilus*. *S*. Typhimurium infection may lead to lower gastrointestinal bleeding [90], while platelet stimulation is crucial in maintaining vascular integrity [91]. The gut is a metabolically active, dynamic organ, and metabolic disturbances can contribute to various intestinal illnesses [92]. Furthermore, the results showed that probiotic supplementation enhanced vital metabolic pathways such as glycolysis, inositol phosphate, and glutathione metabolism, affecting digestion, food absorption, and gut health. The integrity of tight junctions and the intestinal barrier is influenced by metabolic pathways like serine, threonine, glycine, inositol phosphate, and methane metabolism [93].

Furthermore, LC-MS analysis contrasts the groups LAST and CPG to evaluate the impact of probiotics on intestinal metabolites. A total of 929 significantly altered metabolites were mapped to the KEGG metabolic pathways. Among these pathways, branched-chain amino acids (BCCAs), such as leucine, isoleucine, and valine, as well as taurine and aminoacyl-tRNA biosynthesis, PPAR signaling pathway, protein digestion and absorption, and mTOR signaling pathway were significantly enriched (Figure 10C). *Lactobacillus acidophilus* can maintain a healthy intestinal balance by lowering intestinal pH and producing metabolites [39]. Additionally, it can also counteract the activity of enzymes produced by pathogens that convert precursors into carcinogens [94]. BCCAs play a crucial role in fat buildup, insulin resistance, and glutathione production [95]. Taurine and aminoacyl-tRNA biosynthesis are linked to several immune functions [96]. These results are in line those of Yan et al. [97], Tang et al. [98], and Yi et al. [99]. *Lactobacillus* strains were thought to prevent diseases by enhancing anti-inflammatory cytokines via mTOR pathway modulation [100]. In our findings, probiotic *L. acidophilus* administration substantially enriched the mTOR pathway during *Salmonella* infection, which is in parallel to the finding of Wang et al. [101].

## 4. Materials and Methods

### 4.1. Animals and Microorganisms

In this study, lyophilized *Lactobacillus acidophilus* (1.3251) containing 5 × 10^8^ CFU/g was sourced from Tianjin Shengji Group Co., Ltd., Tianjin, Hubei, China. *Salmonella* Typhimurium (CMCC 1.1194) was obtained from the National Center for Medical Culture Collection, Beijing, China. The *Lactobacillus acidophilus* was reconstituted in a 10% skim milk suspension, while *Salmonella* was cultured using selective medium selenite cystine broth and bismuth sulfite agar.

Female, 4–5-week old specific pathogen-free (SPF) BALB/c mice weighing 16–17 g were procured from Bay fu Beijing Biotechnology Co., Limited (Beijing, China). Following their arrival, the mice were provided with a 7-day acclimation period to settle into the laboratory environment. The mice had ad libitum access to food and water throughout the experimental and acclimation phases. They were housed under a 12 h light/dark cycle in a facility maintained at 21 ± 2 °C with a relative humidity of 45 ± 0.1%. To prevent cross-contamination between the treated and untreated groups, they were housed in separate rooms. Furthermore, additional environmental and physical measures were implemented per established standards to mitigate the risk of contamination [102]. All animal experiments and procedures were approved by the Research Ethics Committee of Guangxi University (GXU-2023-0125).

### 4.2. Experimental Design

The experiment comprised three phases: the primary stage (days 1 to 6), the infection phase (day 7), and the last phase (days 8 to 21). Twenty-four mice were divided into four groups (n = 6).

(1) Control negative group (CNG): this group served as the non-treated, non-challenged control, where mice received only normal saline.
(2) Positive control group (CPG): Mice in this group were challenged but not treated. They were fed a standard diet and infected intragastrically via a feeding needle with *S*. Typhimurium on day 7.
(3) *Lactobacillus acidophilus*-fed non-challenged group (LAG): mice in this group received *L. acidophilus* suspended in a 10% skim milk suspension but were not infected with *Salmonella*.
(4) *Lactobacillus acidophilus*-fed infected group (LAST): Mice in this group were fed intragastrically with *L. acidophilus* probiotic suspended in a 10% skim milk suspension at a concentration of 5 × 10^8^ CFU/mL for up to one-week pre-infection. On day 7, they were infected with *S*. Typhimurium using the same intragastric gavage method.

The LD_50_ for *S*. Typhimurium was determined to be 1 × 10^10^ CFU/mL, but we intentionally choose a sublethal dose of 1 × 10^8^ CFU/mL to mimic the human infection. This dose facilitated the close monitoring of behavior, weight loss, immunity, and other physiological responses while enabling the observation of host–pathogen interactions without compromising animal welfare. This aligns with the clinical manifestation of sublethal gastrointestinal symptoms in human infections, thereby enhancing the relevance of our experimental model.

On day 7, the CPG and the LAST were infected intragastrically via a feeding needle with a sublethal dose of 0.2 mL of *S*. Typhimurium suspension containing 1 × 10^8^ CFU/mL. In contrast, mice in the CNG and the LAG were administered normal saline to deliver a similar management stress. Following infection, the LAG and the LAST were treated daily with *L. acidophilus* suspended in a 10% skim milk suspension at a concentration of 5 × 10^8^ CFU/mL via intragastric gavage for two weeks. By cervical dislocation, all mice were euthanized 14 days post-infection.

### 4.3. Health-Related Behavior (General Health Score)

The general health score (GHS) index, ranging from 1 to 5 [103] (Table S1), was employed to assess the health-related behaviors of all groups, particularly the *Salmonella*-challenged groups, on days 1, 7 (day of infection), 14, and 21. Additionally, each group’s average weight and food and water intakes were evaluated on alternate days before and after infection by providing each group with a known amount of food and a known volume of water in their home cages, and then weighing the remaining food and volume of water once per day. Ambulation, grasping reflex (Table S2), and *Salmonella* fecal count tests were also conducted (detailed methods are included in Supplementary File S1).

### 4.4. DNA Isolation and 16srRNA Amplicon Sequencing

Three fresh samples were collected from the intestinal fecal contents (one from each replicate cage) on the 21st day of the experiment, and genomic DNA was isolated using the QIAamp DNA Stool Mini kit (Qiagen, Hilden, Germany). The concentration and integrity of DNA were determined using a Nano-drop 2000 spectro-photometer (Thermo Fisher Scientific, Waltham, MA, USA) and further validated by 1% agarose gel electrophoresis. The V3–V4 hypervariable region of the 16s rRNA gene was amplified by PCR using universal primers 338F-(5′-ACTCCTACGGGAGGCAGCA-3′) and 806R-(GGACTACHVGGGTWTCAAT-3′) [104]. The detailed information on the thermal cycling program is provided in Supplementary File S1.

### 4.5. Sequence Processing, Taxonomy Assignments, and Community Structure Analysis

The Quantitative Insights into Microbial Ecology (QIIME) platform (v1.8.0) was utilized for sequencing data analysis [105]. Sequences effectively matched with barcodes were assigned to specific samples. Low-quality sequences, defined by lengths of less than 50 bp, Phred scores of less than 20, and containing ambiguous bases, were filtered out. By using FLASH, paired-end reads were produced [106]. The remaining sequences were further processed to identify and remove chimeric sequences with UCHIME. Operational taxonomic units (OTUs) were then clustered at 97% similarity using Uparse (v7.0.1001) software [107]. The taxonomic classification of OTUs was performed using the RDP Classifier algorithm in the SILVA database https://www.arb-silva.de/ (accessed on 9 November 2023). Alpha diversities related to community richness and diversity were assessed. Additionally, beta diversity was evaluated by principal coordinate analysis (PCoA) based on unweighted UniFrac distances. Non-metric multidimensional scaling (NMDS) analysis using Bray–Curtis difference matrices was conducted to identify variances and similarities between treated and untreated groups. To explore the influence of treatment on the abundance of bacterial genera, redundancy analysis (RDA) was performed using R (version 3.2.2). The association between genera and ecological variables was examined using the Spearman correlation mantel test. Structural changes in bacterial communities and taxonomic abundances at different levels between groups were statistically obtained using linear discriminant analysis effect size (LEfSe) [108]. For the LEfSe analysis, a threshold LDA score of >2.0 and a threshold ratio of 0.08 were applied. Additionally, mRNA expression levels of different cytokines were assessed. Detailed materials and methods, including the primers used, are discussed in Supplementary File S1 and Table S3.

### 4.6. RNA Extraction for Transcriptome Analysis

On day 14 post-infection, three mice per group were euthanized, and 2 cm-long small intestine tissue samples (ileum) were collected to analyze the transcriptional response of *L. acidophilus* during *Salmonella* infection. Total RNA was isolated using the TRIzol method [109], following the manufacturer’s instructions (Magigene Biotechnology Co., Ltd., Guangzhou, China). The integrity of the RNA was assessed using the Agilent 4200 system (Agilent Technologies, Waldbronn, Germany), and the RNA concentration was determined using NanoDrop One (Thermo Fisher Scientific, Waltham, MA, USA).

### 4.7. Library Construction and Quant 3′ Sequencing for Transcriptomic Analysis

Using 120 ng of RNA, QuantSeq libraries were generated using Lexogen’s QuantSeq 3′ mRNA-Seq Library Prep Kit (Vienna, Austria) for Illumina (San Diego, CA, USA). RT primers, such as oligo-dT with Illumina-compatible 5′ adapters, captured the 3′-end of mRNA during reverse transcription. After the hydrolysis of RNA strands with RNase H-specific hydrolase, a second DNA-RNA hybrid was synthesized using random primers. cDNA fragments (150–200 bp) were isolated using carboxyl-modified GE Sera-Mag Magnetic speed beads. PCR was performed with 2 × PfuMax HiFi PCR ProMix (EnzyValley, Guangzhou, China) and VAHTS Multiplex Oligos Set 4 (Vazyme, Nanjing, China) for Illumina index primers. The PCR products were purified with carboxyl-modified GE Sera-Mag Magnetic Speed beads, and the insert size was determined using the Qsep400 system. The index-coded samples were clustered using the cBot cluster formation technology and then sequenced on the Illumina Novaseq 6000 platform (Illumina San Diego, CA, USA), generating 150 bp paired-end reads.

Raw data were preprocessed using Fastp (v0.23.2) to remove low-quality reads and ensure data accuracy by analyzing Q20, Q30, GC content, and repetitive sequences. Mus musculus reference genomes and annotation files were obtained from https://www.ncbi.nlm.nih.gov/datasets/genome/GCF_000001635.27/ (accessed on 7 November 2023). Alignment was performed using Hisat2 (v2.2.1), and gene read counts were obtained.

### 4.8. Differentially Enriched Metabolites and Functional Enrichment Analysis

For the metabolome analysis, the intestinal fecal contents (100 mg) from the colon were collected and mixed with 400 µL of the extract solution (cold methanol/acetonitrile/water = 2:2:1 *v*/*v*), which contained a stable isotope internal standard. The mixture was vortexed for 30 s, ground for 4 min, and sonicated for 5 min at 35 Hz. Subsequently, it was incubated for 1 h at −40 °C and centrifuged at 12,000 rpm for 15 min at 4 °C. The supernatant was collected and filtered before being detected in a 2 mL injection vial. All samples were combined into QC samples with the same amount of supernatant.

A UHPLC system (Vanquish, Thermo Fisher Scientific) equipped with a UPLC BEH (2.1 mm × 100 mm, 1.7 m) amide column coupled to a Q Exactive HFX mass spectrometer (Orbitrap MS, Thermo) was used for the LC-MS study. The mobile phase was 25 mmol/L ammonium acetate and 25 mmol/L ammonia hydroxide in water (pH 9.75) (A) and acetonitrile (B). The auto-sampler temperature was maintained at 4 °C, and the injection volume was 3 μL. The QE HFX mass spectrometer was employed for its ability to acquire MS/MS spectra in the information-dependent acquisition (IDA) mode under the direction of the acquisition software (Xcalibur 2.1.0, Thermo). The electrospray ionization (ESI) source parameters included a sheath gas flow rate of 30 Arb, an auxiliary gas flow rate of 25 Arb, a capillary temperature of 350 °C, full MS resolution of 60,000, MS/MS resolution of 7500, collision energy set at 10/30/60 in NCE mode, and a spray voltage of 3.6 kV (positive) or −3.2 kV (negative), as appropriate. Differentially enriched metabolites (DEMs) were analyzed based on the criteria that included VIP ≥ 1, *p* ≤ 0.05, and log2FC > 1.5.

## 5. Conclusions

This study emphasizes the pivotal significance of the probiotic *L. acidophilus* (1.3251) in mitigating the adverse effects of *Salmonella* infection. The comprehensive approach examines the microbiome, transcriptome, and metabolome during *Salmonella* infection and *L. acidophilus* treatment. The result confirms that probiotic *Lactobacillus acidophilus* (1.3251) can increase the abundance of the commensal microbial population, which may increase butyrate production, change anti-inflammatory, antioxidant, and metabolite factors, and activate immunological pathways against infections. Changes were observed in essential pathways related to immunity and inflammation, suggesting *L. acidophilus*’s significance in enhancing defense systems. Enriched metabolic pathways highlight its importance in digestion, nutritional absorption, and gut health, including the maintenance of cell structures and tight junctions. The study of intestinal metabolites reveals how they influence lipid metabolism and immunological function. The results provide a baseline for the possible therapeutic use of *L. acidophilus* (1.3251) against infections, but it is important to acknowledge that this study has limitations, especially in regard to animal models and experimental conditions, encouraging caution while extrapolating its results to real-world scenarios. Further study should be conducted to elucidate the complex mechanisms behind *L. acidophilus*’s ability to protect against *Salmonella* and other infections. Furthermore, exploring the potential synergistic effects of *L. acidophilus* supplementation with other probiotics, prebiotics, or antibiotics could provide valuable insights into combination therapies for enhanced efficacy against infections. This holistic approach holds promise for advancing our understanding of probiotic interventions and their role in promoting gastrointestinal health and immunity.

## Data Availability

The study’s datasets, including metadata and raw/processed data, are available in online repositories via the assigned NCBI accession number, i.e., PRJNA1056116.

## Supplementary Materials

The following supporting information can be downloaded at: https://www.mdpi.com/article/10.3390/antibiotics13040352/s1, Table S1. Health-related score index. Table S2. Ambulation test’s scoring parameters. Table S3. List of primer sequences used in this study. Table S4. Descriptive statistical analysis of RNA-seq data. Table S5. Showing important up and down regulated genes related to immunity, metabolism, cell regulation and tight junctions. Table S6. Significantly Enriched upregulated and downregulated GO (BP) terms in LAST versus CPG group. Table S7. KEGG Pathways analysis Of Top 30 Differentially Expressed Genes between LAST VS CPG. Table S8. Showing Significantly up and downregulated Metabolites between LAST versus CPG. Figure S1. Effects of treatments on alpha diversity index (A), Chao-1 index (B), Simpson index (C), and Reads Richness (D) using the Kruskal and Wilcox test, *p* ≤ 0.05. Figure S2. The rare fraction curve for alpha diversity shows enough depth and richness between the groups CNG, CPG, LAG, and LAST. Figure S3. (A) PCoA of intestinal microbiota on the last day of the experiment based on weighted uni-Frac dissimilarity. (B) Non-metric multi-dimensional scaling analysis of the gut microbiota based on the Bray–Curtis distance for CNG, CPG, LAG, and LAST using Anosim (R = 0.042) (*p* = 0.04). For NMDS, the stress value was 0.1, which indicated a good representation. Figure S4. Hierarchal clustering analysis showing differences between the groups CNG, CPG, LAG, and LAST. Figure S5. Intestinal flora biomarkers amongst groups. (A) LEfSe analysis showed differentially abundant taxa produced by the Kruskal–Wallis test. (B) Cladogram of taxa abundances between groups. Taxa lacking significant differences are labeled in yellow, whereas significantly diverse taxa are labeled using the color of the individual group; red color indicates the control positive group (CPG), and green color indicates the treatment group (LAST). Taxa with a log-linear dis-criminant analysis (LDA) score of >2 were finally considered (*p* ≤ 0.05). Figure S6. Intestinal flora biomarkers amongst groups. (A) LEfSe analysis showed differentially abundant taxa produced by the Kruskal–Wallis test. (B) Cladogram of taxa abundances between groups. Significantly diverse taxa are labeled using the color of the individual group; red color indicates the control negative group (CNG), and green color indicates the control positive group (CPG). Taxa with a log-linear discriminant analysis (LDA) score of >2 were finally considered (*p* ≤ 0.05). Figure S7. Intestinal flora biomarkers amongst groups. LEfSe analysis showed differentially abundant taxa produced by the Kruskal–Wallis test. (B) Cladogram of taxa abundances between groups. Taxa lacking significant differences are labeled in yellow, whereas significantly diverse taxa are labeled using the color of the individual group; red color indicates the control negative group (CNG), and green color indicates the probiotic group (LAG). Taxa with a log-linear dis-criminant analysis (LDA) score of >2 were finally considered (*p* ≤ 0.05). Figure S8. (A) Principal component analysis (PCA) showed significant variance between samples. (B) Orthogonal partial least squares discriminant analysis (OLPS-DA). (C) Permutation test. (D) S-plot showed the separation of sample classes based on metabolite profiles between LAST and CPG groups. Figure S9. Hierarchical heatmap clustering analysis of differentially upregulated and downreg-ulated metabolites between LAST and CPG groups. Red color indicates the upregulated metabo-lite, and blue indicates the downregulated metabolite. Figure S10. Ternary plot analysis displaying the enriched and the depleted genera in terms of bacterial community composition between the groups CNG, CPG, and LAG. References [110,111] are cited in the Supplementary Materials.
