# Supplementary material for: Deciphering Microbiome, Transcriptome, and Metabolic Interactions in the Presence of Probiotic Lactobacillus acidophilus against Salmonella Typhimurium in a Murine Model
Source: Antibiotics (Basel). 2024 Apr 11;13(4):352. doi: 10.3390/antibiotics13040352 (PMC11047355; doi:10.3390/antibiotics13040352)
Supplement: Supplementary file 1 [file antibiotics-13-00352-s001.zip › Supplementary File S1.pdf]

Supplementary Material

Deciphering Microbiome, Transcriptome, and Metabolome Interactions in the Presence of Probiotic *Lactobacillus acidophilus* against *Salmonella Typhimurium* in a Murine Model

Muhammad Junaid [1], Hongyu Lu [1], Ahmad Ud Din [3], Bin Yu1, Yu Liu [1], Yixiang Li [1], Kefei Liu [2], Zhong-Quan Qi [1]*, Jianhua Yan [1,]*

1. Medical College, Guangxi University, Nanning 530004, Guangxi, China
2. Tianjin Shengji Group., Co Ltd. No 2, Hai Tai Development 2nd Road, Huayuan Industrial Zone, Tianjin China.
3. Plants for Human Health Institute, Department of Food Bioprocessing and Nutrition Sciences North Carolina State University 600 Laureate Way, Kannapolis, NC 28081, USA

* Correspondence: jianhuayan@gxu.edu.cn; yxyyz@gxu.edu.cn;

1. Materials and Methods

1.1. Health-Related behavior (General Health score)

Table S1. Health-related score index.

	Criteria of Health Index	Score
1	The mouse has bright eyes and is attentive. It has a smooth coat with a shine, reacts to stimuli, and is interested in its surroundings.	5
2	The fur is slightly ruffed, and the coat has lost its lusters, yet the mouse is alert and active.	4
3	Ruffed fur and clumpy coat patches are noticeable, and the animal is less interested in the environment outside the cage.	3
4	Mouse slouched and listless, showing little interest in surroundings, with clumped fur and evidence of hyperventilation when handled.	2
5	The mouse is frigid to touch and non-responsive to stimuli, and its fur has a bottle-brush look.	1

1.2. Ambulation and Grasping Reflex test

The ambulation test assesses balance while walking on a scale of 0 to 3. Mice were positioned to be visible from the top and the side on a rough, white surface. Mice were gently pushed to induce them to walk, and scores were counted according to criteria (Table S2). The scoring method was adapted from Feather-Schussler et al. [1] and performed on day 10 post-infection. Salmonella infections cause imbalance or a loss of grip and grasping power. The mouse was gripped by its neck as if the dam was carrying it. A coarsely shaped plastic card was rubbed across each mouse paw, and the number of paws with which the card was clutched was counted.

Table S2. Ambulation test's scoring parameters.

S.NO	Criteria of Ambulation	Score
1	no movement	0
2	crawling with asymmetric limb movement	1
3	slow crawling but symmetric limb movement	2
4	fast crawling/walking	3

1.3. Salmonella Fecal Count

A total (100 mg) of fresh fecal samples were obtained by gently squeezing the rectal part of the mice aseptically on alternative days, i.e., days 1, 3, 6, 9, 12, and 14 post-infections. The feces were gently homogenized in sterile saline (5 ml) and serially diluted with the same diluent. To enumerate total Salmonella in feces, a total of 0.1 ml of the diluted sample was plated on bismuth sulfite agar, incubated at 37 °C for 24 to 48 hours, and the colony counts were performed. The results were expressed as a $\log_{10}$ cfu/g of feces.

1.4. Thermal Cycling Protocol

The thermal cycling program was composed of an initial 3 minutes of denaturation at 95 °C, followed by 27 cycles of denaturation for 30 s at 95 °C, annealing for 30 s at 55 °C, and extension for 45 s at 72 °C, with a final 7 minutes of extension at 72 °C. Each sample was run in triplicate. The PCR reaction contained 2 µL of 2.5 Mm dNTPs, 4 µL of 5× Fast Pfu buffer, 0.4 µL of Fast Pfu Polymerase, 0.8 µL of each primer, 0.2 µL of BSA, and 10ng of DNA template. The PCR was run in a 2% electrophoresis chamber and then purified using an AxyPrep DNA Gel Extraction Kit (Axygen Bioscience, Union City, CA, USA) per the manufacturer's instructions. The amplicons were used for library preparation and pyrosequencing after being purified. The NEB-Next® Ultra™ DNA Library Preparation Kit (New England Bio-labs, Ipswich, MA, USA) was used to generate the sequencing libraries, and the sequencing of libraries was performed using the Illumina MiSeq PE 300 platform (Illumina, Inc., San Diego, CA, USA).

1.5. Cytokine mRNA Transcript Analysis

After 14 days post-infection, the mice were euthanized under aseptic conditions by cervical dislocation. The small intestine (ileum) 1–2-centimeter fragments in length were obtained and kept in RNAlater, frozen at -80°C for the successive isolation of RNA by the TRIzol method following the manufacturer manual (2306001, Beijing Solarbio Science and Technology Co, Ltd). The RNA was quantified by the means of Nanodrop (ND1000), and a total of 2 µm of RNA was reverse transcribed using a reverse transcription kit, according to the manufacturer's instructions (KR116-01, Tiangen Biochemical Technology (Beijing) Co, Ltd). The PCR reaction was achieved by using optimized conditions: 95 °C for 5 min, followed by 25 cycles of 30 s at 95 °C, 30 s at 60 °C, and 72 °C for 30 s with a 10 min extension at 72 °C. The mRNA expression levels of IL-1α, IL-1β IL-6, TNFα,1L-10, CLDN-1, SOD1, BCL-2, Bax, and caspase-3 were measured by qRT-PCR. The primer sequences used in this experiment are shown in Table S3 of Supplementary Material. β-actin was considered as a reference gene, and the average mRNA expression levels were calculated by the $2^{\Delta\Delta}$Ct method [2].

Table S3. List of primer sequences used in this study.

Genes	Primers (Sequence 5'-3')
IFN-β	Forward-AACTCCACCAGCAGACAGTG
	Reverse-GGTACCTTTGCACCCTCCAG
β-actin	Forward-AGAGAAGCTGTGCTATGTTGCT
	Reverse-GGAACCGCTCGTTGCCAATA
SOD-1	Forward-GCGGATGAAGAGAGGCATGT
	Reverse-TTCCACCTTTGCCCAAGTCA
TNF-α	Forward-GATCGGTCCCCAAAGGGATG
	Reverse-CCACTTGGTGGTTTGTGAGTG
IL-1α	Forward-CGTGTTGCTGAAGGAGTTGC
	Reverse-GGTGCACCCGACTTTGTTCT
IL1-β	Forward-TGCCACCTTTTGACAGTGAT

	Reverse-GTGCTGCTGCGAGATTTGAA
CLDN1	Forward-GGCTTCTCTGGGATGGATCG
	Reverse-TTTGCGAAACGCAGGACATC
BAX	Forward-CTGGATCCAAGACCAGGGTG
	Reverse-GTGAGGACTCCAGCCACAAA
Bcl-2	Forward-CTGGGATGCCTTTGTGGAACT
	Reverse-CAGGTATGCACCCAGAGTGATG
Caspase3	Forward-GGAGCAGCTTTGTGTGTGTG
	Reverse-AGCCTCCACCGGTATCTTCT
IL-6	Forward-AGTTGTGCAATGGCAATTCTGA
	Reverse-TCCAGGTAGCTATGGTACTCCA
IL-10	Forward-GCAAGGGTGTCTCCTTCCTC
	Reverse-CTTGTTACACTCGCCCCCTT

Results

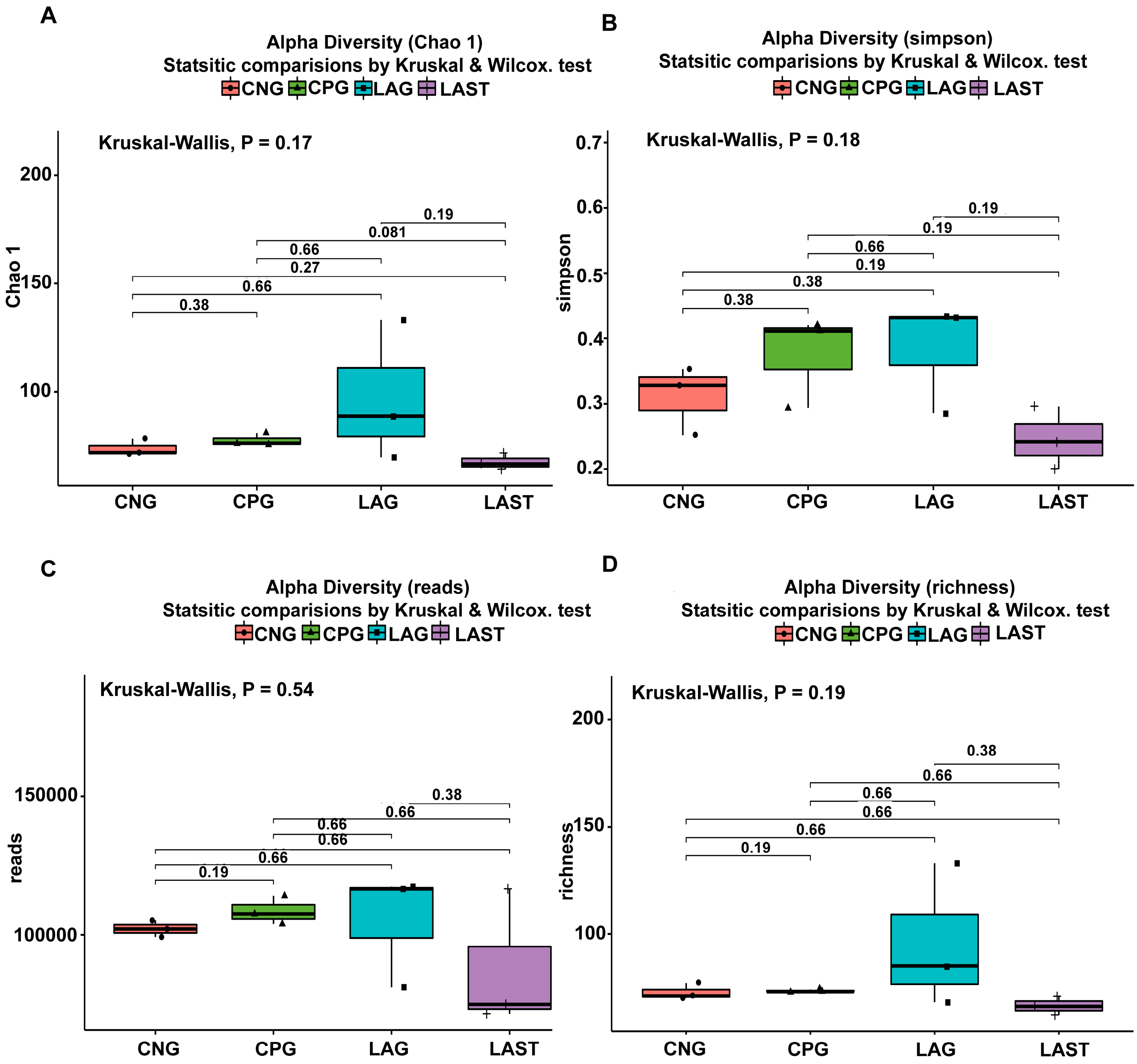

Figure S1. Effects of treatments on alpha diversity index (A), Chao-1 index (B), Simpson index (C), and Reads Richness (D) using the Kruskal and Wilcox test, $P \leq 0.05$.

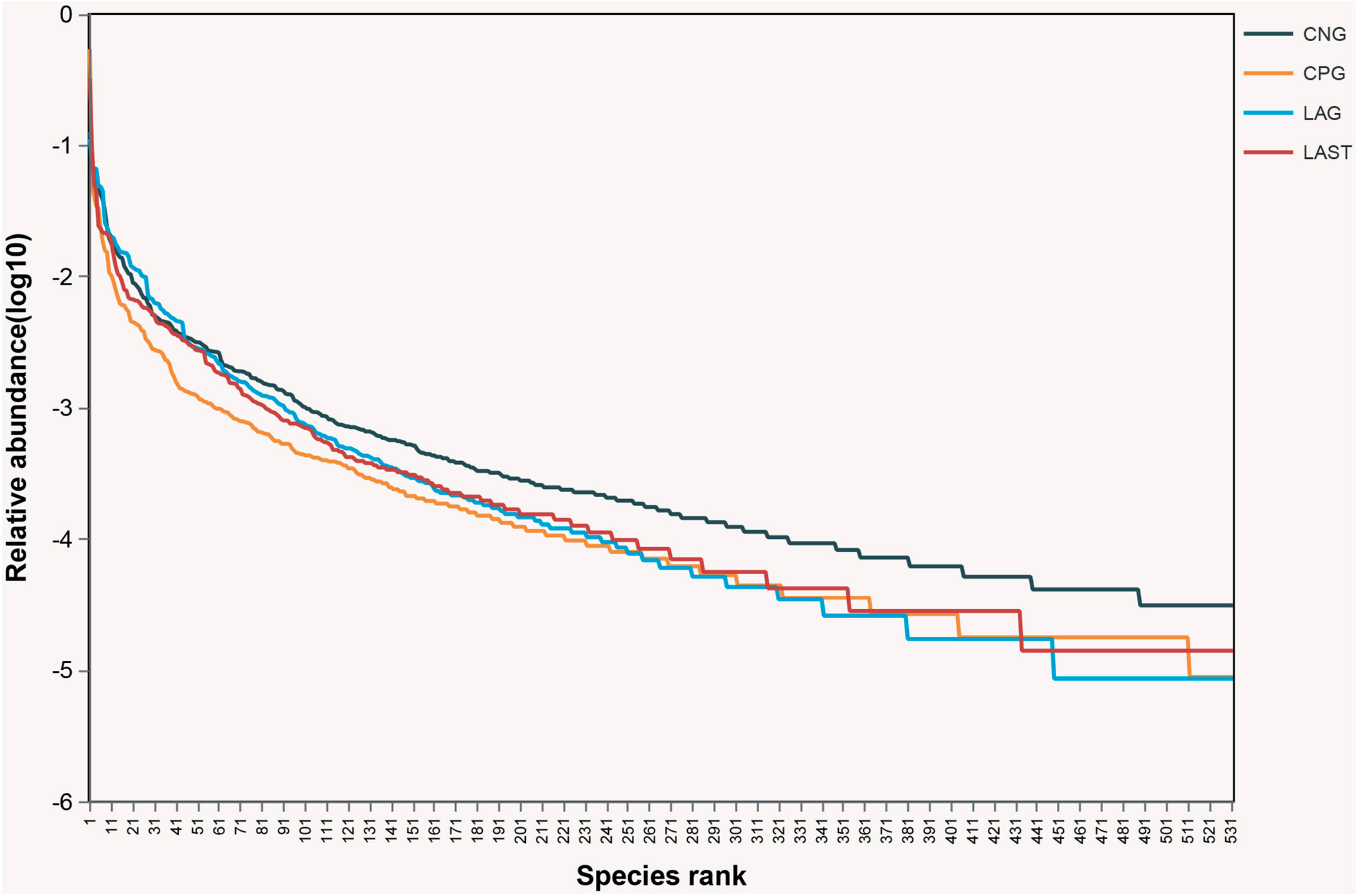

Figure S2. The rare fraction curve for alpha diversity shows enough depth and richness between the groups CNG, CPG, LAG, and LAST.

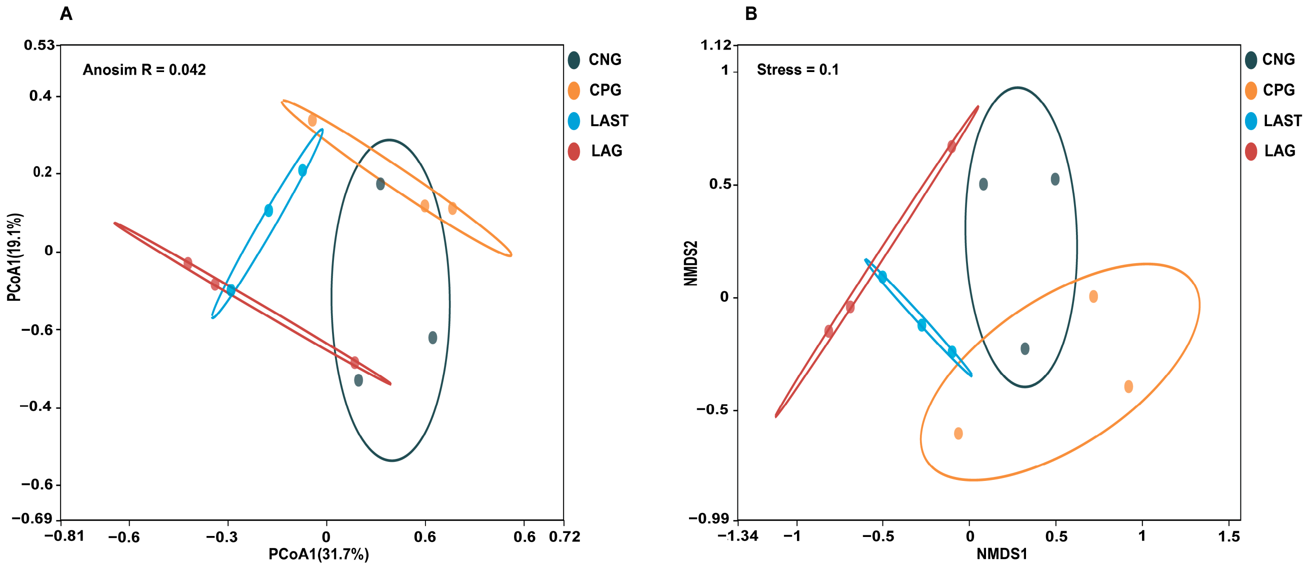

Figure S3. (A) PCoA of intestinal microbiota on the last day of the experiment based on weighted uni-Frac dissimilarity. (B) Non-metric multi-dimensional scaling analysis of the gut microbiota based on the Bray–Curtis distance for CNG, CPG, LAG, and LAST using Anosim (R = 0.042) (p = *0.04*). For NMDS, the stress value was 0.1, which indicated a good representation.

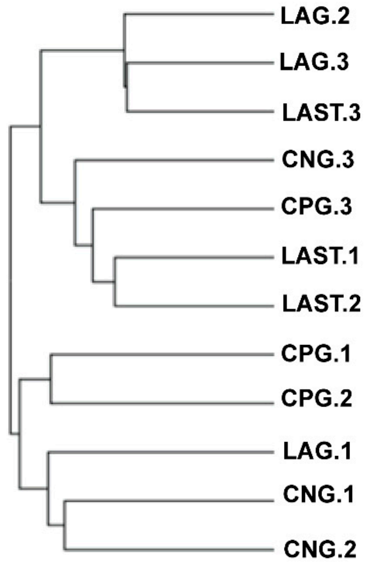

Figure S4. Hierarchal clustering analysis showing differences between the groups CNG, CPG, LAG, and LAST.

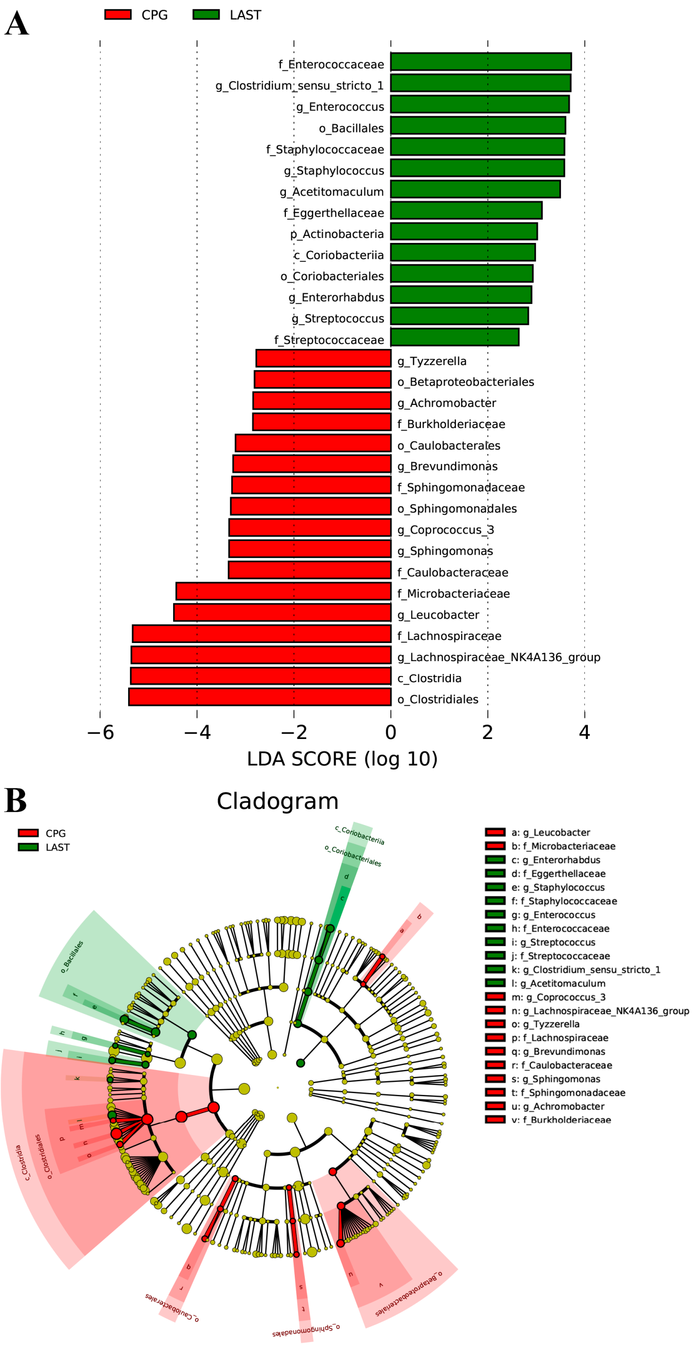

Figure S5. Intestinal flora biomarkers amongst groups. (A) LEfSe analysis showed differentially abundant taxa produced by the Kruskal–Wallis test. (B) Cladogram of taxa abundances between groups. Taxa lacking significant differences are labeled in yellow, whereas significantly diverse taxa are labeled using the color of the individual group; red color indicates the control positive group (CPG), and green color indicates the treatment group (LAST). Taxa with a log-linear discriminant analysis (LDA) score of >2 were finally considered ($P \leq 0.05$).

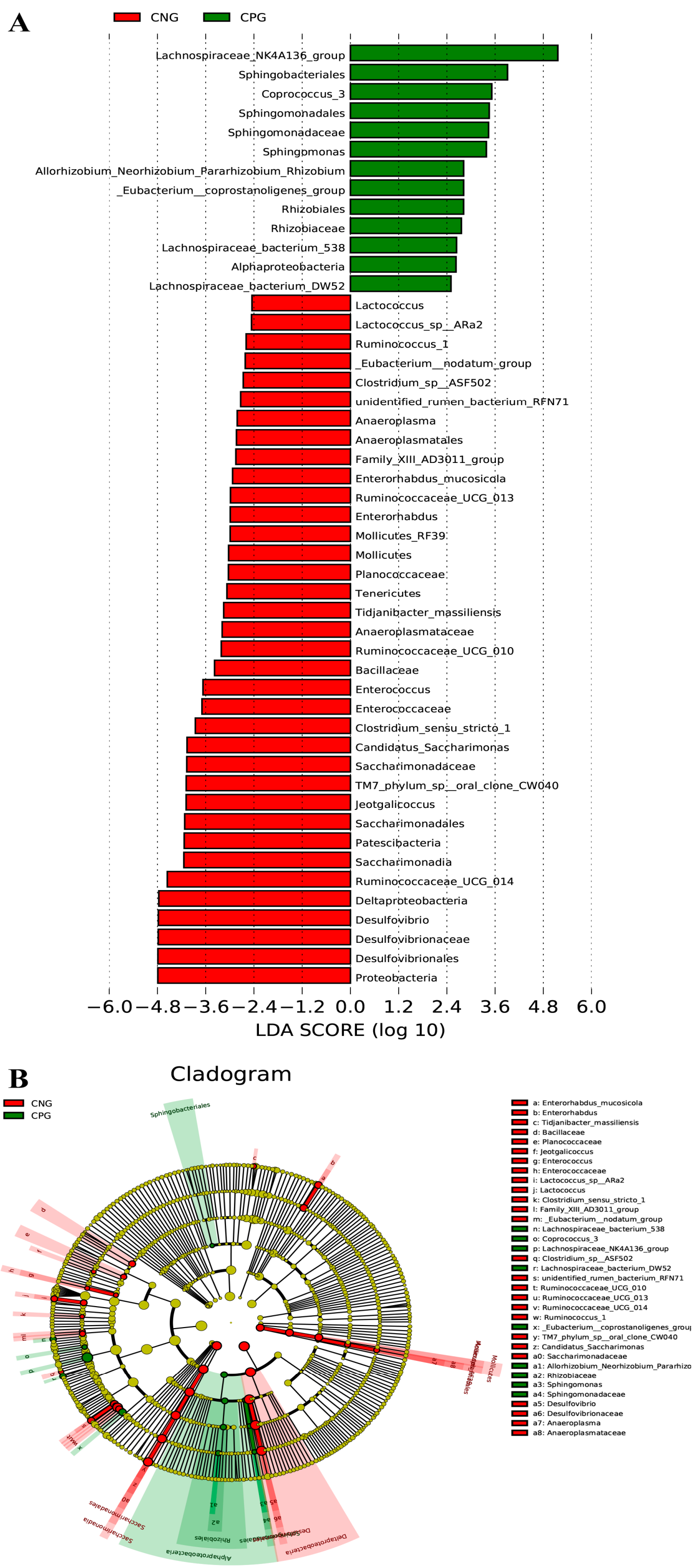

Figure S6. Intestinal flora biomarkers amongst groups. (A) LEfSe analysis showed differentially abundant taxa produced by the Kruskal–Wallis test. (B) Cladogram of taxa abundances between groups. Significantly diverse taxa are labeled using the color of the individual group; red color indicates the control negative group (CNG), and green color indicates the control positive group (CPG). Taxa with a log-linear discriminant analysis (LDA) score of >2 were finally considered (*P ≤ 0.05*).

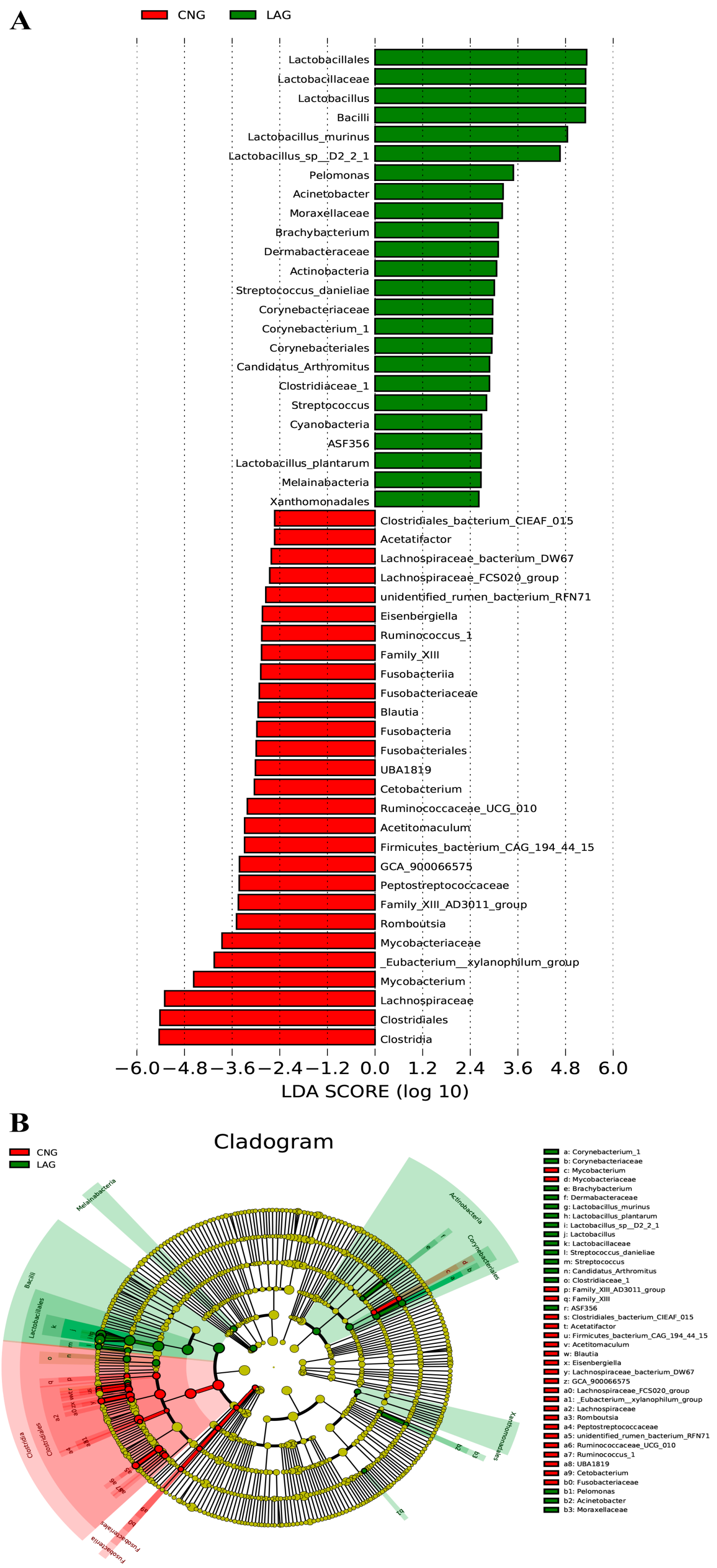

Figure S7. Intestinal flora biomarkers amongst groups. LEfSe analysis showed differentially abundant taxa produced by the Kruskal–Wallis test. (B) Cladogram of taxa abundances between groups. Taxa lacking significant differences are labeled in yellow, whereas significantly diverse taxa are labeled using the color of the individual group; red color indicates the control negative group (CNG), and green color indicates the probiotic group (LAG). Taxa with a log-linear discriminant analysis (LDA) score of >2 were finally considered ($P \leq 0.05$).

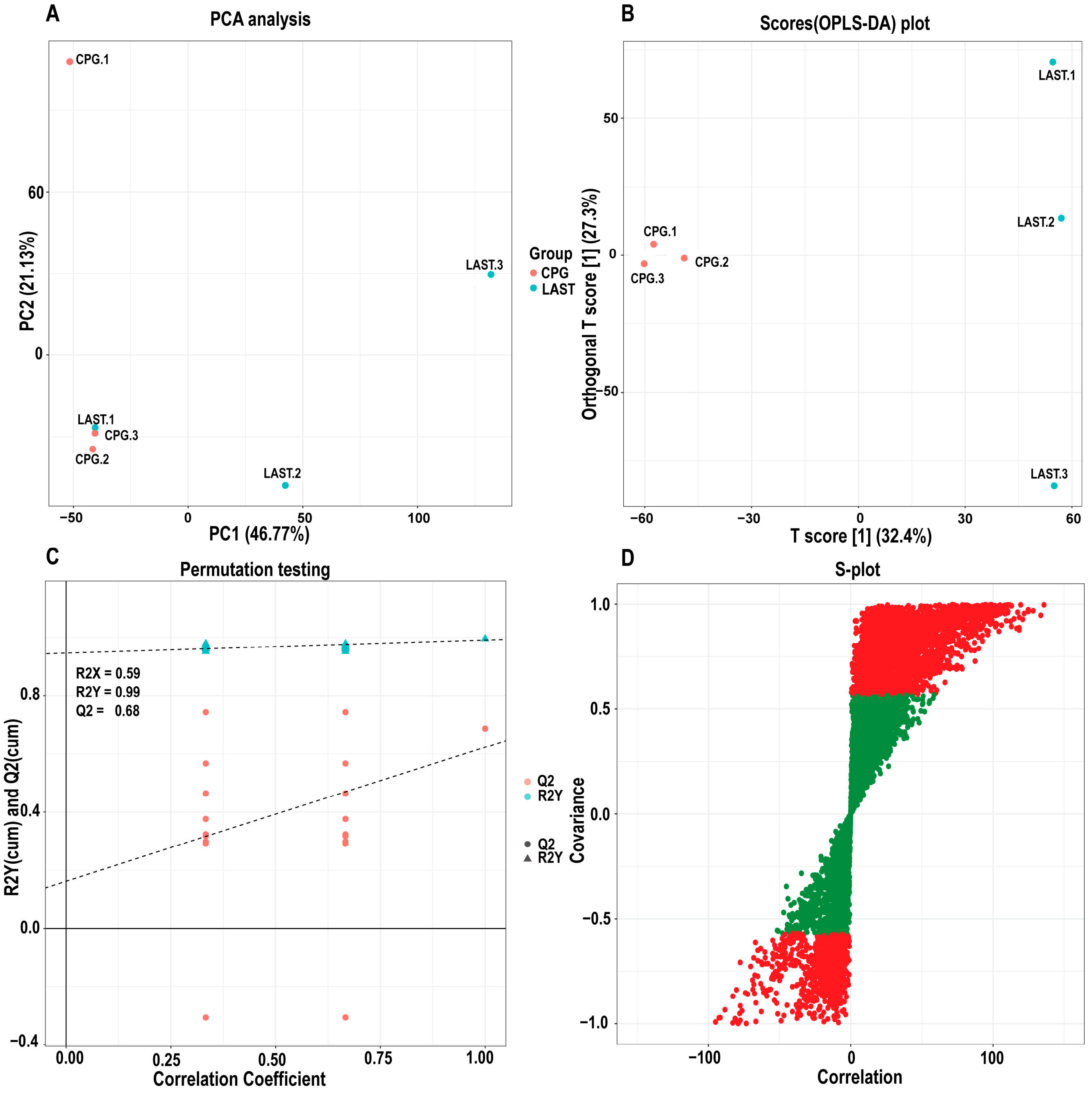

Figure S8. (A) Principal component analysis (PCA) showed significant variance between samples. (B) Orthogonal partial least squares discriminant analysis (OLPS-DA). (C) Permutation test. (D) S-plot showed the separation of sample classes based on metabolite profiles between LAST and CPG groups.

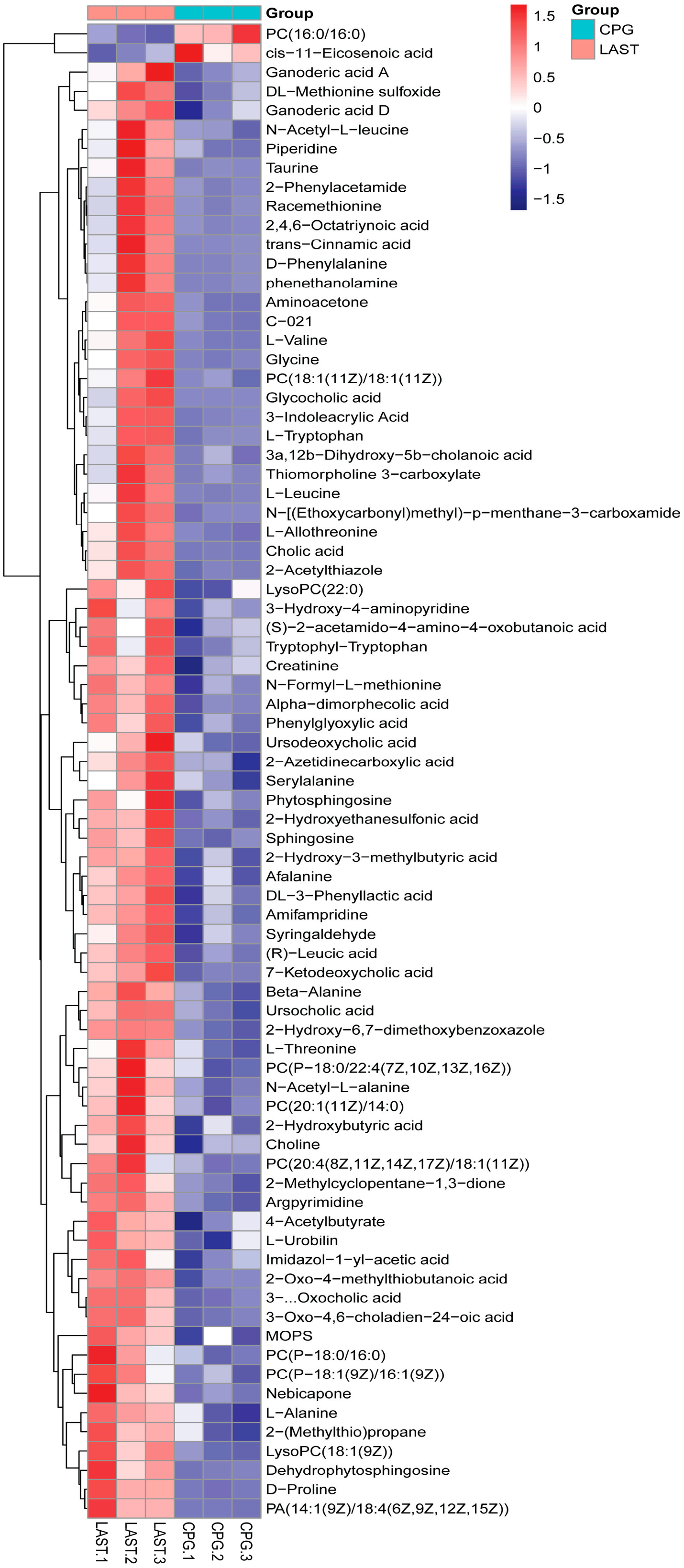

Figure S9. Hierarchical heatmap clustering analysis of differentially upregulated and downregulated metabolites between LAST and CPG groups. Red color indicates the upregulated metabolite, and blue indicates the downregulated metabolite.

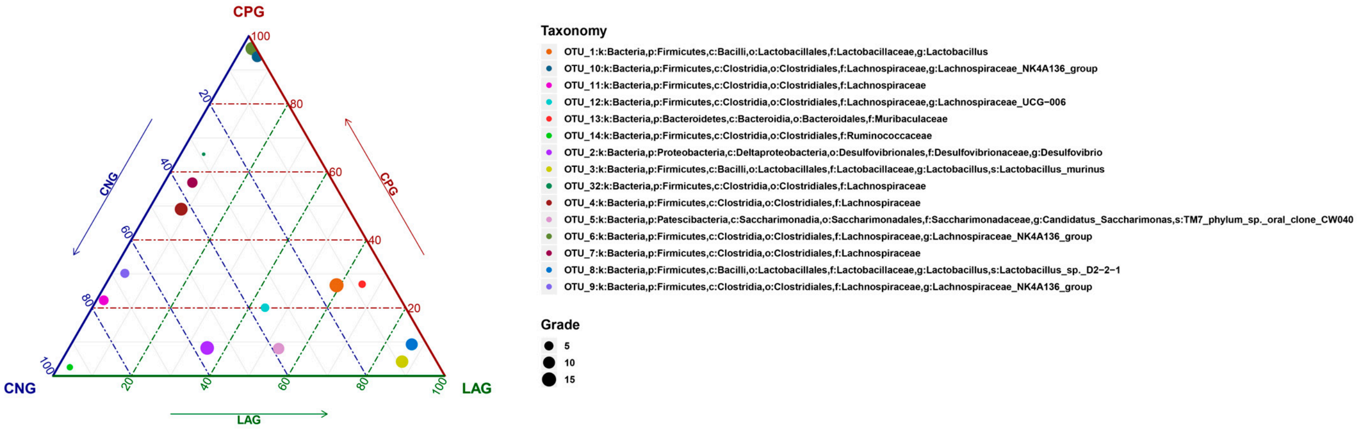

Figure S10. Ternary plot analysis displaying the enriched and the depleted genera in terms of bacterial community composition between the groups CNG, CPG, and LAG.

References

1. Feather-Schussler, D.N. and T.S.J.J.o.v.e.J. Ferguson, *A battery of motor tests in a neonatal mouse model of cerebral palsy.* 2016(117).
2. He, Y., et al., *Protection of surface layer protein from Enterococcus faecium WEFA23 against Listeria monocytogenes CMCC54007 infection by modulating intestinal permeability and immunity.* Applied Microbiology and Biotechnology, 2021. **105**(10): p. 4269-4284.
